# *APETALA 2‐*like genes *AP2L2* and *Q* specify lemma identity and axillary floral meristem development in wheat

**DOI:** 10.1111/tpj.14528

**Published:** 2019-10-15

**Authors:** Juan Manuel Debernardi, Julian R. Greenwood, E. Jean Finnegan, Judy Jernstedt, Jorge Dubcovsky

**Affiliations:** ^1^ Department of Plant Sciences University of California Davis CA 95616 USA; ^2^ Howard Hughes Medical Institute Chevy Chase MD 20815 USA; ^3^ CSIRO Agriculture and Food GPO Box 1700 Canberra ACT 2601 Australia

**Keywords:** *Triticum aestivum*, *Triticum turgidum*, spikelet development, floral meristem, miRNA, AP2, floral organs, lodicules

## Abstract

The spikelet is the basic unit of the grass inflorescence. In tetraploid (*Triticum turgidum*) and hexaploid wheat (*Triticum aestivum*), the spikelet is a short indeterminate branch with two proximal sterile bracts (glumes) followed by a variable number of florets, each including a bract (lemma) with an axillary flower. Varying levels of miR172 and/or its target gene *Q* (*AP2L5*) result in gradual transitions of glumes to lemmas, and vice versa. Here, we show that *AP2L5* and its related paralog *AP2L2* play critical and redundant roles in the specification of axillary floral meristems and lemma identity. *AP2L2*, also targeted by miR172, displayed similar expression profiles to *AP2L5* during spikelet development. Loss‐of‐function mutants in both homeologs of *AP2L2* (henceforth *ap2l2*) developed normal spikelets, but *ap2l2 ap2l5* double mutants generated spikelets with multiple empty bracts before transitioning to florets. The coordinated nature of these changes suggest an early role of these genes in floret development. Moreover, the flowers of *ap2l2 ap2l5* mutants showed organ defects in paleas and lodicules, including the homeotic conversion of lodicules into carpels. Mutations in the miR172 target site of *AP2L2* were associated with reduced plant height, more compact spikes, promotion of lemma‐like characters in glumes and smaller lodicules. Taken together, our results show that the balance in the expression of miR172 and *AP2‐*like genes is crucial for the correct development of spikelets and florets, and that this balance has been altered during the process of wheat and barley (*Hordeum vulgare*) domestication. The manipulation of this regulatory module provides an opportunity to modify spikelet architecture and improve grain yield.

## Significance Statement

A better understanding of wheat spike development is essential to engineer varieties that are more productive. This study shows that wheat *APETALA 2*‐like genes *AP2L2* and *Q* (= *AP2L5*), which are targets of miR172, play essential and redundant roles in floret development. These genes have additional effects on heading time, plant height, spikelet and floret number, and lodicule development. We identified two induced *AP2L2* alleles resistant to miR172 that can be used to modify these traits in wheat.

## Introduction

Cereal inflorescence architecture is a major determinant of grain yield and, not surprisingly, it has been extensively modified by human selection during crop domestication. Modifications in cereal inflorescence development facilitated increases in grain number and size, and fine‐tuned factors limiting shattering while improving threshability (Doebley, 2006; Debernardi *et al*., 2017). A better understanding of the molecular mechanisms that control inflorescence development may allow the engineering of new architectures with enhanced grain productivity.

Inflorescence development begins when the shoot apical meristem (SAM) transitions from the vegetative to the reproductive phase. In the ancestral grass inflorescence, the panicle, the inflorescence meristem (IM) generates lateral primary and secondary branches, each ending in a spikelet (‘little spike’). In tetraploid (*Triticum turgidum*) and hexaploid wheat (*Triticum aestivum*), the primary and secondary branches of the inflorescence are absent, resulting in spikelets attached directly to the rachis, forming a derived structure called a spike. The wheat IM generates a determined number of lateral spikelet meristems (SMs) in an alternating distichous pattern along the central rachis before forming a terminal spikelet (Kellogg *et al*., 2013).

The spikelet is the basic unit of the grass inflorescence and comprises a series of overlapping bracts arising distichously from a short axis called the rachilla (Clifford, 1987). In wheat, the two proximal bracts lack axillary meristems and are designated as glumes, whereas the next bracts, called lemmas, have axillary meristems that develop into short reproductive shoots. In the floral axis, the floral meristem generates the palea (a membranous two‐keeled structure), two scales called lodicules that can swell to spread the lemma and palea, three stamens and a terminal ovary. These lateral shoots with their subtending lemmas are designated as florets (Clifford, 1987). In some grass species the SM produces a determinate number of florets, e.g. in *Hordeum vulgare* (barley), *Oryza sativa* (rice), *Sorghum *sp. and *Zea mays* (maize), whereas wheat generates an indeterminate number of lateral florets with only the most basal florets surviving to support grains (Kellogg, 2001; Guo *et al*., 2017; Sakuma *et al*., 2019).

The grass inflorescence architecture is determined by the maintenance or termination and the identities acquired by the IM and lateral meristems, which in turn depend on the expression and interactions of developmental regulatory genes in the meristem or in adjacent signaling centers (Whipple, 2017; Bommert and Whipple, 2018). In wheat, it was recently shown that the MIKC‐type MADS‐box proteins of the APETALA 1 (AP1)‐like family (VRN1, FUL2 and FUL3) play central roles controlling the activity and determinacy of the IM and the specification of the SMs and their subtending bracts (Li *et al*., 2019). These and other MIKC‐type MADS‐box proteins play conserved roles in the specification of SM fate and floral organ identity, which are well documented in the ABCDE model of flower development (Callens *et al*., 2018; Wu *et al*., 2018; Chongloi *et al*., 2019). MADS‐box genes act as tetrameric complexes and different protein combinations result in the specification of different floral organ identities (Theissen *et al*., 2016).

In contrast, the mechanisms and genes that control the transition of the SM, from producing sterile glumes to florets, are not entirely clear. Members of the *APETALA 2* (*AP2*)*‐*like family of transcription factors (TFs) are good candidates for this function. Combined mutations in two closely related *AP2‐*like genes from maize, *INDETERMINATE SPIKELET 1* (*IDS1*) and *SISTER OF INDETERMINATE SPIKELET 1* (*SID1*) (Chuck *et al*., 2008), or in the two orthologs from rice, *OsIDS1* and *SUPERNUMERARY BRACT* (*SNB*) (Lee and An, 2012), result in spikelets that generate multiple bract‐like structures before producing one or more florets (except for the maize tassel, where no florets are formed). In polyploid wheat, the orthologs of *IDS1* include the well‐known gene *Q* on chromosome 5A. This gene played a critical role during wheat domestication by conferring the square spike and free‐threshing characteristics (Simons *et al*., 2006). Loss‐of‐function mutants in the *Q* gene in tetraploid wheat also resulted in the formation of additional sterile bracts with characteristics intermediate between glumes and lemmas (Debernardi *et al*., 2017).

Studies of the conserved microRNA172 (miR172), which targets *AP2‐*like mRNAs for cleavage (Huijser and Schmid, 2011; Zhu and Helliwell, 2011), provide additional evidence for the roles of *AP2*‐like genes in inflorescence and spikelet development. In maize, disruption of the miR172e locus by a transposon insertion in the mutant *tasselseed 4* (*ts4*) or a single mutation within the miR172 binding site of *IDS1* (*Ts6* allele) resulted in the production of additional florets in the spikelet and a lack of pistil abortion in the tassel (Chuck *et al*., 2007). Similarly, disruption of barley miR172 by a transposon insertion showed abnormal spikelet development, including the conversion of glumes to partially developed florets in apical regions of the spikes (Brown and Bregitzer, 2011). The domesticated *Q* allele in wheat originated from a point mutation in the miR172 target site that reduced miR172 activity. Further reduction in miR172 activity (generated by target mimicry, henceforth MIM172) or an extra point mutation in the miR172 binding site of *Q* showed a similar conversion from glumes to florets in spikelets located in distal positions of the spike (Debernardi *et al*., 2017; Greenwood *et al*., 2017). In spikelets located in subterminal positions, glumes were converted into sterile florets consisting of only a lemma and a palea. In proximal spikelets, the glumes subtended no axillary meristems but had longer awns and reduced keels relative to the wild type, indicative of a partial transition to lemmas. This gradient of homeotic conversions correlated with a decrease of miR172 and an increase of *Q* expression levels from basal to apical regions of the spike (Debernardi *et al*., 2017).

**Figure 1:**
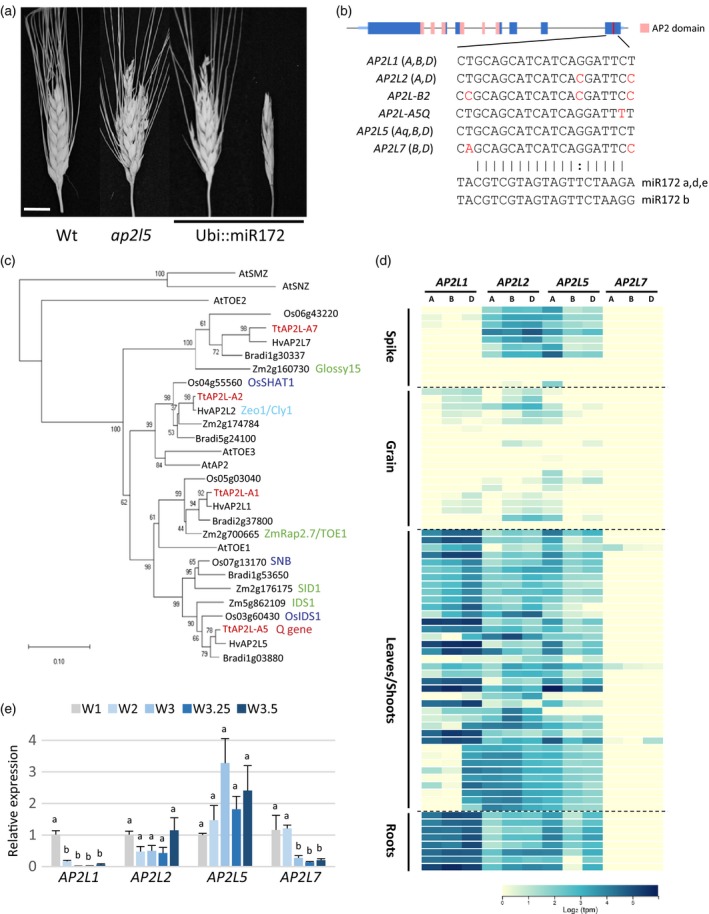
Wheat *AP2*‐like genes. (a) Representative spikes of a wild‐type plant (Wt), *ap2l5* null mutant and T_0_ transgenic lines transformed with a vector expressing the miR172d precursor under the maize *UBIQUITIN* promoter (Ubi::miR172). Scale bar: 1 cm. (b) Genomic structure of a typical *AP2*‐like gene, showing the exons in blue, the AP2 domains in pink and the miR172 target site in red (sequences for different wheat *AP2*‐like genes below, with variants in red). (c) Neighbor‐joining molecular phylogenetic analysis of Arabidopsis, *Brachypodium*, rice, maize, barley and wheat AP2‐like proteins (based on alignments of the two AP2‐domains). (d) RNA‐seq heat map showing the expression of different *AP2*‐like homeologs from hexaploid wheat in different tissues (data from the web tool available at http://www.wheat-expression.com). Each row corresponds to a different developmental stage (from younger to older) of the tissue listed on the left. (e) Relative expression of *AP2*‐like genes in apices from the tetraploid variety Kronos at different developmental stages on the Waddington scale (W1, vegetative apex; W2, early double‐ridge stage; W3, glume primordium; W3.25, lemma primordium; W3.5, floret primordium). Expression data were determined by qRT‐PCR using the ΔΔ*C*_t_ method and *ACTIN* as endogenous control. Expression was normalized to W1 stage for each gene. Bars represent means ± SEMs of three or more biological replicates, and different letters indicate statistically significant differences (*P *<* *0.05).

Overexpression of an miR172 precursor driven by the maize *UBIQUITIN* promoter (Ubi::miR172) also results in alterations in inflorescence and spikelet development. Rice Ubi::miR172 panicles showed reduced branching and additional glume‐like bracts similar to those observed in *snb osids1* double mutants. The Ubi::miR172 plants with the highest miR172 expression levels showed more severe effects than the double mutants, however, suggesting that additional miR172 targets were likely involved in the regulation of these phenotypes (Lee and An, 2012). A similar result was observed in wheat transgenic plants overexpressing miR172. Most wheat Ubi::miR172 plants showed similar phenotypes to the *Q‐*null mutants, with one or two florets transformed into sterile glume‐like structures (Debernardi *et al*., 2017). However, two out of the 14 independent wheat transgenic plants showed an even stronger phenotype, with a large number of sterile glume‐like organs (Figure 1a). These plants failed to produce seeds, limiting further analyses and precluding their inclusion in our previous study.

We hypothesized that the more severe spikelet phenotypes observed in the strong Ubi::miR172 plants relative to the wheat *Q‐*null and rice *snb osids1* double mutants (Lee and An, 2012) could result from the downregulation of additional *AP2‐*like paralogs. In this study, we combined gene expression data with transgenic and genetic approaches to show that another *AP2‐*like gene, an ortholog to barley *HvAP2 Cly1/Zeo* and rice *SHAT1* genes (Nair *et al*., 2010; Zhou *et al*., 2012; Houston *et al*., 2013; Wang *et al*., 2015a), plays an important and overlapping role with *Q* in wheat floret development.

## Results

### Wheat *AP2*‐like family

To prioritize which mutant *AP2‐*like genes to combine with the *Q* loss‐of‐function mutant, we characterized the A, B and D homeologs for the three other wheat *AP2*‐like genes harboring a miR172 target site (Figure 1b; Table S1; from Wheat Genome RefSeqv1.1). We designated these genes as *AP2L1*,* AP2L2*,* AP2L5* (synonymous with *Q*) and *AP2L7*, with numbers corresponding to their chromosome locations. Henceforth, and to avoid confusion, we will use the symbols *Q* and *q* to refer specifically to the A‐genome alleles with or without the mutation in the miR172 binding site, respectively, *AP2L5* when referring to the overall function of the different homeologs, and *ap2l5* when referring to the loss‐of‐function mutants for all homeologs.

A phylogenetic analysis including all *AP2*‐like genes targeted by miR172 from wheat, barley, rice, maize, *Brachypodium* and Arabidopsis showed that the wheat *AP2L5* gene belongs to the *IDS1/SID1* cluster (Figure 1c), and that the ortholog of *SID1/SNB* is absent in wheat and barley genomes. We also failed to detect an ortholog of *SID1/SN*B in the available genomic sequences of *Secale cereal* (rye), *Triticum urartu* (einkorn wheat, an A‐genome progenitor), *Aegilops tauschii* (a D‐genome progenitor) and *Triticum turgidum* ssp. *dicoccoides* (wild emmer), accession Zavitan (Avni *et al*., 2017) (Table S2). These observations suggest that the ortholog of *SID1*/*SNB* was probably lost in the tribe Triticeae.

The wheat gene most closely related to *AP2L5* is *AP2L1*, a homolog of the flowering repressor *TOE1* from Arabidopsis and maize (Aukerman and Sakai, 2003; Salvi *et al*., 2007). The RNA‐seq data available (Borrill *et al*., 2016; Ramirez‐Gonzalez *et al*., 2018) show that *AP2L1* is expressed at very low levels in the spike (Figure 1d), making it an unlikely candidate for inflorescence or flower development. Wheat *AP2L*7, the ortholog of maize *GLOSSY15* (Moose and Sisco, 1996) (Figure 1c), was nearly undetectable in all tissues, whereas *AP2L2* showed a very similar expression profile to *AP2L5*, with high transcript levels in the spikes (Figure 1d). Wheat *AP2L2* belongs to the same clade as the rice *SHAT1*, barley *Cly1/Zeo1* and Arabidopsis *AP2* genes (Jofuku *et al*., 1994; Nair *et al*., 2010; Zhou *et al*., 2012; Houston *et al*., 2013) (Figure 1c), which are all important regulators of inflorescence and/or flower development.

To confirm the published RNA‐seq data, we performed quantitative reverse transcription PCR (qRT‐PCR) on cDNA derived from the vegetative SAM and early stages of spike development identified using the Waddington scale (Waddington *et al*., 1983) (Figure 1e). All four *AP2‐*like genes were expressed in vegetative apices (W1), but *AP2L1* and *AP2L7* expression decreased after the reproductive transition (from W2 to W3.5). Expression of *AP2L5* and *AP2L2* did not change significantly in the different developmental stages and were expressed at the floret primordia stage (W3.5), when floral meristems and floral organs are specified (Figure 1e). Based on the expression data and the phylogenetic proximity to other *AP2*‐like genes involved in inflorescence development, we prioritized *AP2L2* for further functional characterization.

### *AP2L2* and *AP2L5* function redundantly in the specification of lemma identity and the development of axillary floral meristems

Using a sequenced mutant population in the tetraploid wheat variety Kronos (Krasileva *et al*., 2017), we identified 100 and 79 mutations in the coding regions of *AP2L‐A2* and *AP2L‐B2*, respectively. For the A‐genome copy, we selected line K2233 that has a mutation in the splicing donor site of the fourth intron, and for the B‐genome copy we selected line K3634 with a mutation in the splicing acceptor site of the fourth intron (Figure S1a). Sequencing of K2233 *ap2l‐A2* transcripts revealed that the splice site mutation causes the use of a nearby intronic GT site, resulting in four additional nucleotides and a frame‐shift mutation (Figure S1b). The encoded protein (393 amino acids) lacks the two critical AP2 domains and is most likely not functional. Transcripts from the K3634 *ap2l‐B2* allele were not detected in the expression experiments (see Experimental procedures), suggesting that the mutation may affect transcript stability. We backcrossed the individual mutant lines with wild‐type Kronos twice to reduce background mutations, and then intercrossed the *ap2l‐A2* and *ap2l‐B2* mutants to generate an *ap2l2‐*null mutant, which for simplicity will be referred to hereafter as *ap2l2*.

**Figure 2:**
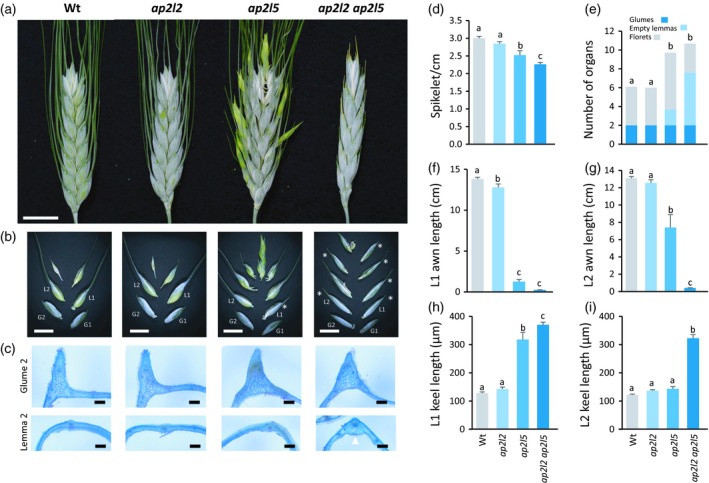
*ap2l2 ap2l5* mutant spikelets produce multiple glume‐like lemmas instead of florets. (a–d) Representative pictures showing the phenotypes observed in the primary spike of Kronos (Wt), *ap2l2*,* ap2l5* and *ap2l2* *ap2l5* mutants. (a) Primary spike 3 weeks after heading. Scale bar: 1 cm. (b) Mature central spikelets with separated organs to show the higher number of empty glume‐like lemmas (white asterisks) in the *ap2l2* *ap2l5* mutant. Scale bar: 1 cm. (c) Transverse sections of the second glume and the second lemma from central spikelets. A white arrowhead points to the more pronounced keel in the second lemma of the *ap2l2 ap2l5* mutant. Scale bar: 200 μm. (d) Spikelet density in the primary spike. (e–i) Number of organs (*n* ≥ 20) (e), length of the awn (*n* ≥ 10) and keel (*n* ≥ 8) in the first (f and h) and in the second (g and i) lemma in the central spikelet of the primary spike. Bars represent means ± SEMs and different letters indicate statistically significant differences (*P* < 0.05) by the Student–Newman–Keuls mean comparison test.

We next compared the phenotypes of *ap2l2* and *ap2l5* mutants in a growth‐chamber experiment. As previously observed, the spikes of the *ap2l5* mutant plants displayed reduced spikelet number and density and a higher number of florets per spikelet than wild‐type spikes (Figure 2a,b,d,e). In contrast, *ap2l2* mutant plants produced spikes that were no different from wild‐type spikes, both in spikelet density and in number of florets (Figure 2a,b,d,e). We observed that the *ap2l2* mutants produced a reduced number of grains per spike (Figure S1c), although we did not detect clear developmental defects in floret organs to explain this observation.

The most important result was observed when we combined the *ap2l2* and *ap2l5* mutations. The *ap2l2 ap2l5* mutant plants (four homozygous mutations) displayed more severe spike phenotypes than the single‐gene mutants (Figure 2a), which were reminiscent of the strongest Ubi::miR172 overexpression plants (Figure 1a). The spikelet density of *ap2l2 ap2l5* was significantly reduced relative to *ap2l5* (Figure 2d), although the spikelet number per spike was unchanged (Figure S2a). Furthermore, the spikelets of *ap2l2 ap2l5* produced an increased number of organs, but instead of florets, we observed mostly empty bracts with no axillary floral organs (Figure 2a,b,e).

In mature spikes of Kronos and the *ap2l2* mutant, glumes had short awns and strong keels, whereas lemmas had elongated awns and less pronounced or no keels (Figure 2b,c). In the spikelets of the *ap2l5* mutant, the first lemma looked like a third glume, as it was always empty, and it had a shorter awn and a more pronounced keel than the wild‐type lemma (Figure 2b,c,f,h). The second lemma had a longer awn, reduced keel and most of the time developed an axillary meristem. In the spikelets of *ap2l2 ap2l5*, all the empty lemmas resembled glumes (Figure 2b), with significantly shorter awns and longer keels than in the *ap2l5* mutant (Figure 2b,c,f–i).

**Figure 3:**
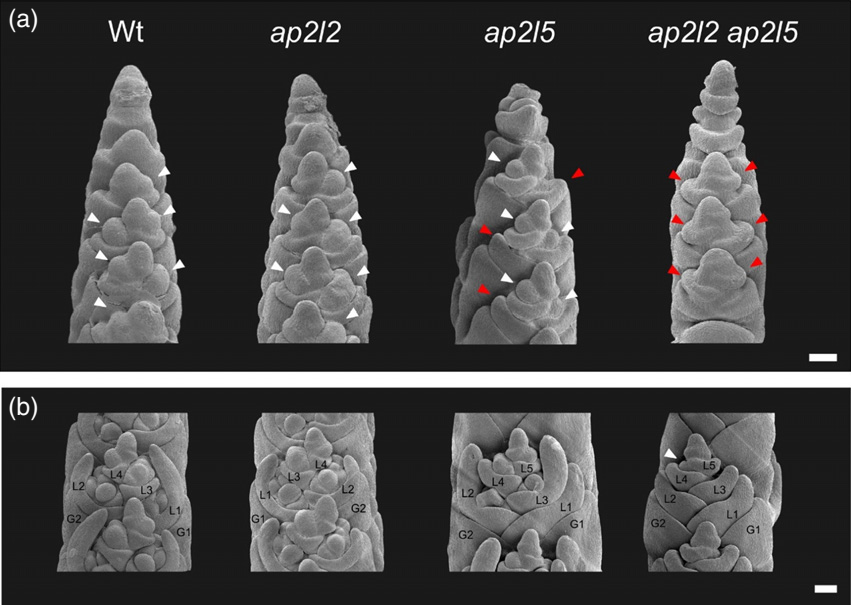
*ap2l2 ap2l5* mutants fail to specify floral meristems during spikelet development. Scanning electron microscopy (SEM) of developing spikes of wild‐type Kronos (Wt), *ap2l2*,* ap2l5* and *ap2l2 ap2l5* mutants. (a) Lateral view of developing spikes collected 24 days after planting (W3.25–W3.5). White arrowheads indicate floral meristems. Red arrowheads indicate empty lemmas. (b) Developing central spikelets from plants 27 days after planting (W3.5–W4.25). At this stage, the wild‐type (Wt), *ap2l2* and *ap2l5* lemmas have elongating awn primordia and surround the growing floral organs. Note that the first lemma (L1) of the *ap2l5* mutant is empty. In the spikelets of the *ap2l2* *ap2l5* mutant most of the lemmas were empty, except for a developing floral meristem surrounded by the fourth lemma (L4, white arrowhead). G1, glume 1; G2, glume 2; L1–L5, lemmas 1–5. Scale bar: 0.5 mm.

To describe the phenotypes in more detail, we dissected and compared immature reproductive apices from the different genotypes (Figures 3 and S3). At the double‐ridge stage, we did not observe differences between the wild type and the mutants (Figure S3a); however, differences became evident during the differentiation of the floral meristems (Figure 3). Scanning electron microscopy (SEM) of the wild type and *ap2l2* showed the normal development of glumes and lemmas with their axillary floral meristems (Figure 3a). In the *ap2l5* mutants, the developing floral meristems were also visible, but the first floret primordium was always replaced by a lemma primordium without an axillary meristem. This phenotype was more severe in the developing spikelets of *ap2l2 ap2l5*, where all initial lemma primordia lacked axillary meristems (Figure 3a). At a later developmental stage, we observed developing floral organ primordia in the spikelets of wild‐type and single‐mutant plants (Figure 3b). At this stage, the *ap2l2 ap2l5* spikelets contained mostly empty bracts, although we observed a floral meristem developing in the axil of some of the late‐developing lemmas (Figure 3b). In a more advanced developmental stage (Figure S3b), the awns of the lemmas were elongated in the wild type, *ap2l2* and *ap2l5* spikes, but not in the *ap2l2 ap2l5* mutant, where the lemma primordia were similar to glume primordia.

Taken together, our phenotypic observations indicate that both *AP2L2* and *AP2L5* promote the transition from glumes to lemmas and the formation of floral meristems in the axil of the lemmas in the developing spikelets.

### *AP2L2* and *AP2L5* regulate floral organ identity and modulate the expression of floral organ identity genes

**Figure 4:**
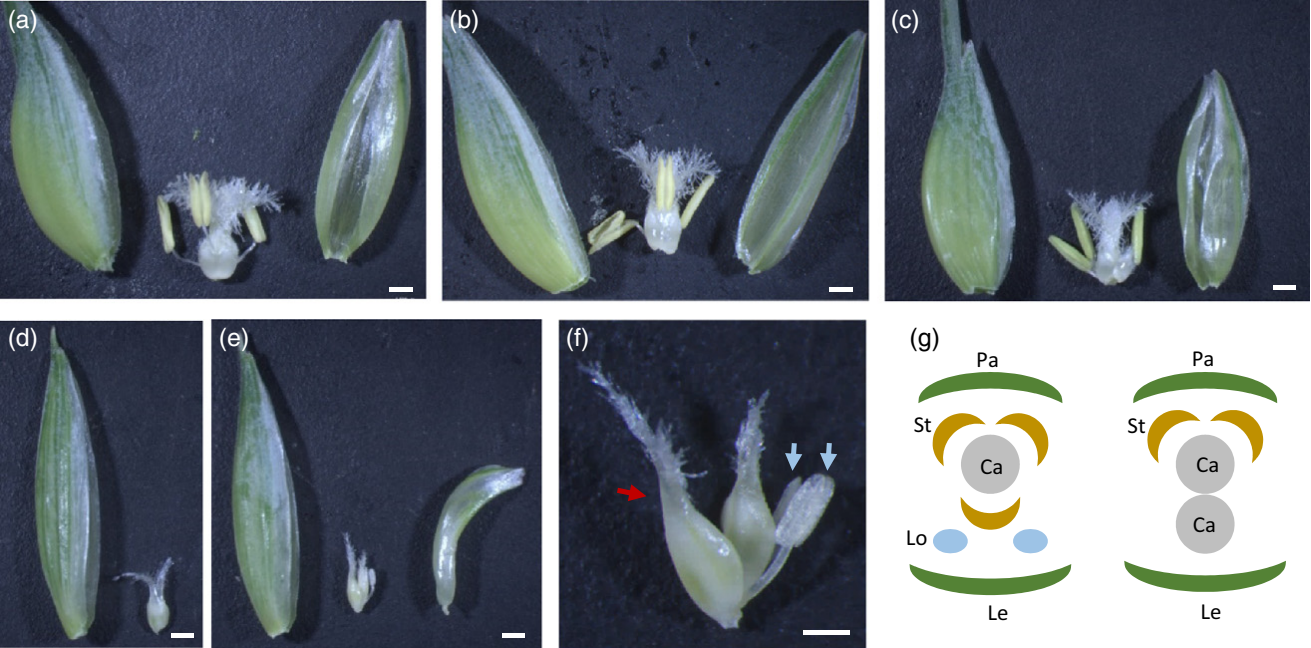
*AP2L2* and *AP2L5* control floral organ identity. (a) Wild‐type first floret from a central spikelet. The lemma and palea were separated to show the inner floral organs. Scale bar: 2 mm. (b) First floret from an *ap2l2* mutant central spikelet. Scale bar: 2 mm. (c) First floret from an *ap2l5* mutant central spikelet. Scale bar: 2 mm. Note that in this mutant background the first floret is generated after one or two glume‐like lemmas are produced. (d–f) Representative florets from *ap2l2 ap2l5* mutants. Scale bar: 2 mm. (f) Magnification of (e) to show the floral organs. Scale bar: 0.5 mm. A red arrow points to a carpelloid organ replacing the lodicules, and white arrows point to immature stamens. (g) Floral diagrams for the wild type (left) and the *ap2l2 ap2l*5 mutant (right). Ca, carpel; Le, lemma; Lo, lodicule; Pa, palea; St, stamen. Note the replacement of lodicules and the adjacent stamen by a carpel.

The conversion from florets to sterile glume‐like lemmas in *ap2l2* *ap2l5* spikes was not complete, and most spikelets were still able to develop axillary flowers, generally in the distal positions of the spikelets (Figure 2e). Those flowers exhibited many developmental defects and did not produce grains, however. Flowers from the wild‐type plants include a carpel surrounded by three stamens, two lodicules and one palea, all subtended by one lemma (Figure 4a,g), whereas in the *ap2l2* *ap2l5* florets the lodicules were absent, the number of stamens was reduced, and occasionally the palea was missing (Figure 4d–f). Interestingly, we observed that in ~40% of the flowers a carpelloid organ replaced the lodicules and the adjacent anterior stamen (Figure 4e–g). In addition, we also observed that ~45% of the flowers developed only carpel‐like structures (Figure 4d). The frequency of these changes in organ number per flower is presented in Table 1 (note that empty glume‐like lemmas were not included in this analysis). Similar phenotypes, but at a lower frequency, were observed in the flowers of the *ap2l5* spikelets (Table 1). The flowers of *ap2l2* had a normal number of organs (Figure 4b), but with larger lodicules compared with wild‐type plants (Figure S1d–f).

**Table 1:** Percentage of plants with different numbers of floral organs per floret in wild‐type (Wt) plants, and in *ap2l2*,* ap2l5* and *ap2l2 ap2l5* mutants. 0 indicates absence. For *ap2l5* and *ap2l2 ap2l5*, empty lemmas were not considered as florets and were not included in the analysis. More than 35 florets from central spikelets were analyzed for each genotype

No. of organs/ floret		0	1	2	3	4	5
Lemma	Wt		100				
	*ap2l2*		100				
	*ap2l5*		100				
	*ap2l2 ap2l5*		100				
Palea	Wt		100				
	*ap2l2*		100				
	*ap2l5*	2.7	97.3				
	*ap2l2 ap2l5*	35.3	64.7				
Lodicules	Wt			100			
	*ap2l2*			100			
	*ap2l5*	21.6	8.1	70.3			
	*ap2l2 ap2l5*	100					
Stamen	Wt			2.5	97.5		
	*ap2l2*			2.9	97.1		
	*ap2l5*	2.7		21.6	64.9	10.8	
	*ap2l2 ap2l5*	44.1	16.2	27.9	10.3	1.5	
Carpel	Wt		100				
	*ap2l2*		100				
	*ap2l5*		91.9	8.1			
	*ap2l2 ap2l5*	1.5	20.6	41.2	26.5	7.4	2.9

**Figure 5:**
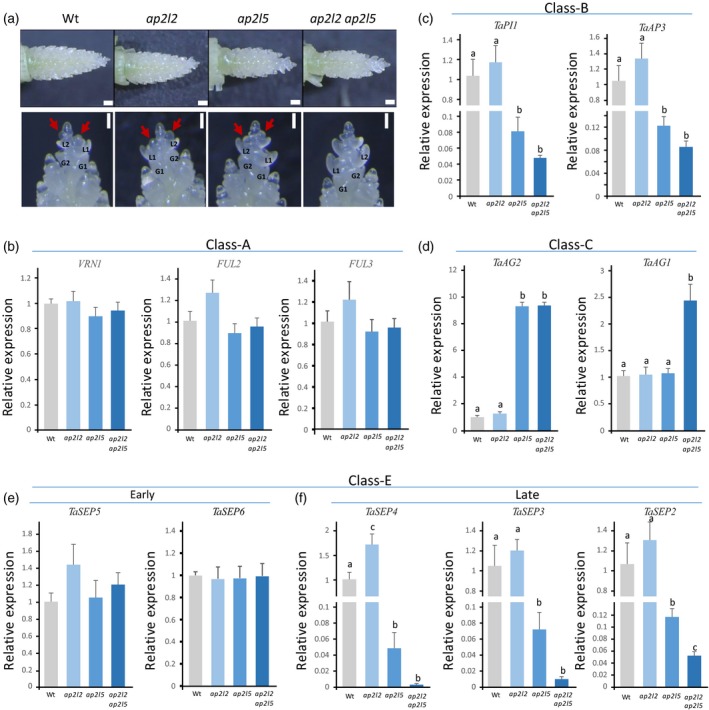
Expression analysis of MIKC‐type MADS‐domain genes of the ABCE flowering model in developing spikes of the wild type (Wt), and the *ap2l2*,* ap2l5* and *ap2l2 ap2l5* mutants. (a) Dissected apices from Wt and *ap2l2*,* ap2l5* and *ap2l2 ap2l5* mutants at the W3.5–W4.25 stage (Waddington scale). Scale bars: 250 μm. Magnified developing terminal spikelets at the W3.5 stage are shown (bottom row). Scale bars: 200 μm. The red arrows indicate floral meristems. (b–f) Transcript levels relative to the *ACTIN* gene. (b) A‐class genes *VRN1*,* FUL2* and *FUL3*. (c) B‐class genes *TaPI1* and *TaAP3*. (d) C‐class genes *TaAG2* and *TaAG1*. (e) Early expressed E‐class genes *TaSEP5* and *TaSEP6*. (f) Late expressed E‐class genes *TaSEP2*,* TaSEP3* and *TaSEP4*. The expression data were determined by quantitative reverse transcription PCR and normalized against the wild type. Bars represent means ± SEMs of four biological replicates, and different letters above error bars indicate statistically significant differences (*P *<* *0.05).

To further describe the mutant phenotypes, we measured the expression of the wheat orthologs of several MIKC‐type MADS‐box genes previously described as members of the ABCE flowering model (Paolacci *et al*., 2007; Theissen *et al*., 2016; Chongloi *et al*., 2019) in the different genotypes by qRT‐PCR (Table S3). We extracted RNA from developing spikes when the lemma, palea and floral meristem primordia were visible at the terminal spikelet (Figure 5a, W3.5–W4.25). As a reference, we compiled the expression levels of the same genes during spikelet development from a published RNA‐seq study (Li *et al*., 2018) and summarized the data in Figure S4.

*VRN1*,* FUL2* and *FUL3*, which belong to the A‐class genes, are the earliest to be expressed and their expression increases through spike development, except for *VRN1* that decreases after the double‐ridge stage (Figure S4a). B‐class *TaPI1* and *TaAP3* (Figure S4b) and C‐class genes *TaAG1* and *TaAG2* (Figure S4c) are both expressed mainly after the glume primordium differentiation stage. Finally, E‐class genes can be divided into two groups based on their expression profiles, an earlier group including *TaSEP5* and *TaSEP6* that is expressed at the double‐ridge stage (Figure S4d) and a later expressing group including *TaSEP2*,* TaSEP3* and *TaSEP4*, which is upregulated at or after the glume primordium differentiation stage (Figure S4e).

We then compared the expression levels of the same genes between Kronos, *ap2l2*,* ap2l5* and *ap2l2 ap2l5* mutants. No significant differences in expression among genotypes was detected for the A‐class genes *VRN1*,* FUL2* and *FUL3* (Figure 5b). By contrast, transcript levels of the B‐class genes were significantly reduced in *ap2l5* and in *ap2l2 ap2l5*, with the latter showing a stronger reduction (Figure 5c). The C‐class gene *TaAG2* was upregulated in *ap2l5* and *ap2l2* *ap2l5*, whereas *TaAG1* was only upregulated in *ap2l2* *ap2l5* (Figure 5d). The early expressing *TaSEP5* and *TaSEP6* showed no significant differences in expression among genotypes (Figure 5e), whereas the late expressing *TaSEP2*,* TaSEP3* and *TaSEP4* were significantly downregulated in *ap2l5* and *ap2l2* *ap2l5*, with the latter showing a stronger reduction (Figure 5f).

Taken together, the expression results described above show that the young spikes of *ap2l5* *ap2l2*, and less dramatically the *ap2l5* single‐gene mutant, have reduced expression of genes involved in floral organ identity (B‐class and late‐expressing E‐class genes), but increased expression of *AG*‐like genes (C‐class).

### Mutations in the miR172 target site of *AP2L2* result in pleiotropic effects on plant height, spike architecture and lodicule size

**Figure 6:**
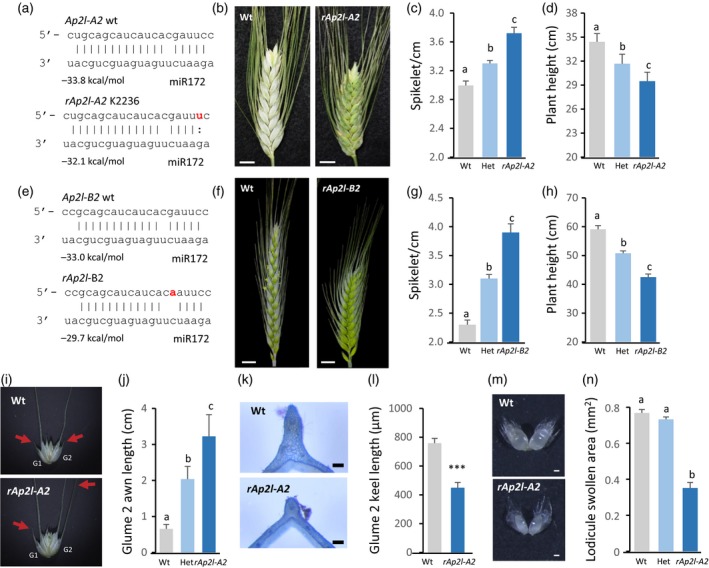
Mutations in the miR172 target site of *AP2L2* (*rAp2l2*) alter spike and floret development and plant height. (a–d) *rAp2l‐A2* in Kronos K2236. (a) Schematic representation of the interaction between miR172 and *AP2L‐A2* miR172 target sites in the wild type and *rAp2l‐A2* (mutation in red). The predicted energy of the interactions is indicated beneath the sequences. (b) Primary spike of a homozygous wild‐type plant (left) and a homozygous *rAp2l‐A2* plant (right), 3 weeks after heading. Scale bar: 1 cm. (c–d) Spikelets per cm in the primary spike (c) and plant height (d) in the F_2_ population segregating for *rAp2l‐A2* (*n* ≥ 8). (e–h) *rAp2l‐B2* in hexaploid wheat Wedgetail. (e) Schematic representation of the interaction between miR172 and *AP2L‐B2* wild‐type and mutant miR172 target sites (mutation in red). The predicted energy of the interactions is indicated beneath the sequences. (f) Primary spike of a wild‐type plant (left) and a homozygous *rAp2l‐B2* plant (right). Scale bar: 1 cm. (g) Spikelets per cm in the primary spike and (h) plant height in cm in an F_2_ population (*n* ≥ 17). (i–j) *rAp2l‐A2* in Kronos K2236. (i) Typical penultimate spikelets from a wild‐type plant and a homozygous *rAp2l‐A2* plant. Red arrows point to glume‐1 (G1) and glume‐2 (G2) awn tips. Scale bars: 1 cm. (j) Length of the awn in the F_2_ population segregating for *rAp2l‐A2* (*n* ≥ 8). (k) Transverse sections and (l) length of the keel of the second glumes in the penultimate spikelet from segregating homozygous wild‐type and *rAp2l‐A2* plants (*n* = 15). (m) Dissected lodicules from homozygous wild‐type and mutant florets. Scale bar: 0.5 mm. (n) Area of the swollen region of lodicules from wild‐type, heterozygous and homozygous *rAp2l‐A2* florets (*n* = 40). Bars represent means ± SEMs. Different letters indicate statistically significant differences (*P *<* *0.05) by the Student–Newman–Keuls mean comparison test. ***Significant difference (*P *<* *0.001) by Student's *t*‐test.

After testing the effect of the *ap2l2* loss‐of‐function mutations in Kronos, we explored the effect of mutations in the miR172 binding site of *AP2L2* in tetraploid Kronos and in hexaploid wheat. First, we identified a tetraploid wheat tilling mutant (K2236) with a point mutation in the miR172 target site of the *AP2L‐A2* homeolog (henceforth resistant *Ap2l‐A2* or *rAp2l‐A2*), in exactly the same position as the one that generated the *Q* allele in *AP2L‐A5* (Figures 6a and 1b). This mutation is silent but significantly affects the repression mediated by miR172 (Debernardi *et al*., 2017). We backcrossed K2236 with wild‐type Kronos and genotyped and phenotyped the F_2_ segregating population. F_2_ plants homozygous for the *rAp2l‐A2* allele had more compact spikes (Figure 6b,c) and were 14% shorter than the wild type (Figure 6d). Plants heterozygous for the K2236 mutation showed intermediate spikelet density and plant height.

A second induced mutation in the miR172 target site of *AP2L2* was identified in the hexaploid winter wheat variety Wedgetail. We had previously identified a dwarf compact spike mutant in a mutagenized population of this variety (Figure 6f). Sequencing of the miR172 target site of the *AP2L‐A5* and *AP2L‐D5* homeologs did not reveal any polymorphisms (*AP2L‐B5* is not functional). Sequencing of the miR172 target site of the *AP2L2* homeologs revealed an SNP at a different position in the miR172 target site of the *AP2L‐B2* gene (henceforth *rAp2l‐B2*, Figure 6e) , however. This mutation produces an Asp→Asn change in the encoded protein, which is predicted to have limited effect on protein structure and function (BLOSUM 62 score = 1). This mutation is also predicted to have a stronger effect on miR172 activity than the mutation previously described for K2236, as reflected by a higher free energy of interaction (Figure 6e). The mutant line was backcrossed with the wild‐type parental line and the F_2_ population was genotyped and phenotyped. F_2_ plants homozygous for the *rAp2l‐B2* allele showed increased spikelet density (even higher than the K2236 mutant; Figure 6f,g) and a 28% reduction in plant height (Figure 6h), both of which co‐segregated with the *rAp2l‐B2* mutant allele.

Reducing miR172 activity in wheat by a MIM172 approach or by mutations in the miR172 target site of *AP2L‐A5* promoted glume‐to‐lemma transitions that were recognized by reduced keels and increased awn length, and in the distal spikelets by the formation of axillary flowers in the glumes (Debernardi *et al*., 2017; Greenwood *et al*., 2017). Interestingly, distal spikelets of the spike of the F_2_ plants carrying the *rAp2l2* alleles have glumes with longer awns and reduced keel compared with the lines carrying the wild‐type alleles both in tetraploid Kronos (Figure 6i–l) and in hexaploid wheat (Figure S5a–c).

In barley, point mutations in the miR172 target site of the *AP2L2* ortholog reduce lodicule swelling (Nair *et al*., 2010), so we examined the lodicules in the wheat F_2_ populations. Lodicules in the tetraploid and hexaploid plants carrying the *rAp2l2* allele were still able to swell at anthesis (Figures 6m and S5d). A detailed inspection showed that the swollen area of the lodicules in the plants carrying the *rAp2l2* alleles was significantly reduced (45% in *rAp2l‐A2* and 30% in *rAp2l‐B2*) when compared with the wild type (Figures 6n and S5d), however. These results indicate that the regulation of *AP2L2* expression by miR172 is important for lodicule swelling in wheat, as was observed in barley.

Taken together, the results from two independent mutant lines show that mutations in the miR172 site of *AP2L2* homeologs induce phenotypes that are similar to the effects generated by the domesticated *Q* allele (reduced plant height, compact spike and glume‐to‐lemma transitions), with the exception of the specific effects on lodicules.

## Discussion

Wheat *AP2L2* and *AP2L5* genes have overlapping roles in the regulation of homeotic changes between glumes and lemmas, the development of axillary floral meristems, spike compactness and plant height. The simultaneous absence of both genes results in spikelets with multiple sterile bracts that form a few distal florets with no lodicules and with other altered floral organs. In addition to their overlapping functions, *AP2L2* affects lodicule size and *AP2L5* affects spikelet number, heading time and floret number. We discuss first the traits for which both genes have overlapping effects and then the specific effects of each gene.

### *AP2L2* and *AP2L5* reduce plant height and increase spike compactness

Mutations in the miR172 binding site of *AP2L2* resulted in reduced plant height in tetraploid and hexaploid wheat, with a stronger effect in hexaploid wheat. This stronger effect is likely to be associated with the more disruptive effect of the *rAp2l‐B2* mutation in hexaploid wheat relative to the *rAp2l‐A2* mutation in tetraploid wheat. An induced mutation in the miR172 binding site of *AP2L‐A5*, which already had the *Q* mutation, also resulted in a severe reduction in plant height (Greenwood *et al*., 2017). These results suggest that both *AP2L2* and *AP2L5* have overlapping roles in the regulation of plant height in wheat. This function seems to be conserved in other grass species because rice MIM172 plants with increased levels of *AP2‐*like genes also exhibited reduced plant height (Wang *et al*., 2015a). The molecular mechanisms responsible for the height changes remain unknown, however.

Both *ap2l2* and *ap2l5* single mutants showed more lax spikes (with a lower number of spikelets per cm), but the differences were significant only for *ap2l5*. However, the *ap2l2 ap2l5* mutant showed a significant reduction in spikelets per cm relative to the *ap2l5* mutant (Figure 2), confirming that both genes have overlapping roles in the regulation of this trait. The role of *AP2L2* in this trait was further demonstrated by the increase in spikelets per cm in *rAp2l2* mutants (Figure 6). Similar observations have been made in barley, where synonymous and non‐synonymous mutations in the miR172 binding site result in spikes that were more compact (Houston *et al*., 2013).

### *AP2L2* and *AP2L5* promote floret development

Homologies between grass‐specific spikelet organs and floral organs in non‐grass species have been widely debated and controversies persist. Glumes are generally interpreted as bracts, but lemmas have been interpreted either as floral bracts or as sepals (Clifford, 1987; Prasad *et al*., 2001; Malcomber *et al*., 2006). Comparative studies and mutants are helping to resolve this controversy. In many grass species, sterile lemmas (with no axillary meristem) are located between glumes and lemmas, suggesting a developmental gradient between these two organs (Malcomber *et al*., 2006). A similar gradient has been observed in tetraploid wheat *ap2l5* mutants and transgenic plants overexpressing miR172. These plants have one or two empty lemmas between the glumes and the fertile lemmas, which are not observed in the wild‐type plants (Debernardi *et al*., 2017). The *ap2l5* mutant also showed lemmas subtending a palea without other floral organs between the empty and fertile lemmas, representing an additional intermediate state (Debernardi *et al*., 2017). The number of empty bracts with intermediate glume‐lemma characteristics increased dramatically in the *ap2l2 ap2l5* mutant, indicating that both genes play redundant roles in these transitions (Figure 2b). By contrast, overexpression of *Q* (Song *et al*., 2019), mismatches in the miR172 binding site of *Q* (Greenwood *et al*., 2017) or increased expression of multiple *AP2‐*like genes by a MIM172 approach resulted in the conversion from glumes to empty or fertile lemmas (Debernardi *et al*., 2017). The continuous and gradual modifications of the morphological characteristics that differentiate glumes from lemmas support the interpretation that glumes and lemmas are homologous organs.

In addition to the different levels of *AP2L5* detected among genotypes, Debernardi *et al*. (2017) observed an miR172 and *AP2L5* gradient along the wheat‐spike axis. The lower levels of *AP2L5* at the base of the spike were associated with a more complete conversion of the lemmas into glumes and a higher proportion of empty lemmas than in more distal parts of the spike (Debernardi *et al*., 2017). The strong correlation between the degree of differentiation of the axillary meristem and the differentiation of its subtending bract suggests that the *AP2L5* and *AP2L2* genes play an early role in floret development, probably before the differentiation of the axillary meristem and its subtending bract. By contrast, rice mutants have been identified that affect the glume–lemma transition (Prasad *et al*., 2001; Yoshida *et al*., 2009, 2012; Gao *et al*., 2010; Hong *et al*., 2010; Lin *et al*., 2014; Wang *et al*., 2017; Wu *et al*., 2018) and the differentiation of the axillary meristem (Zhang *et al*., 2017) separately.

Previous results in other grass species also support an important role of *AP2‐*like genes in the development of the axillary meristems. Double mutants in the maize *AP2‐*like genes *ids1 sid1* fail to produce florets but generate many bract‐like structures in the tassel (Chuck *et al*., 2008). In rice, *osids1 snb* mutants also exhibited spikelets with multiple glume‐like structures, but eventually produced floral organs. An even more severe phenotype was observed in rice plants overexpressing miR172, which suggests that additional miR172 target genes participate in the determination of the axillary floral meristem (Lee and An, 2012). In this study, we show that *AP2L2*, a paralog distinct from *SID1/SNB* (Figure 1c), has an overlapping role with *AP2L5* in the regulation of the floral axillary meristems, and that *ap2l2 ap2l5* mutants have multiple glume‐like organs that failed to produce axillary floral meristems. This phenotype was very similar to the strongest wheat Ubi::miR172 lines, which suggests that *AP2L2* and *AP2L5* account for most of the effect of the miR172‐targeted genes involved in this function. We speculate that mutations in the rice *AP2L2* ortholog *OsSHAT1* combined with *osids1* *snb* would result in a phenotype similar to that observed in the rice transgenic plants overexpressing miR172 (Lee and An, 2012). By contrast, a reduction of miR172 activity in wheat, barley and maize promoted the formation of axillary floral meristems in the spikelets (Chuck *et al*., 2007; Debernardi *et al*., 2017; Greenwood *et al*., 2017; Zhao *et al*., 2018), suggesting a negative and conserved role of miR172 in the specification of axillary floral meristems.

*AP2‐*like genes in both grasses and Arabidopsis share a common role in promoting the establishment and differentiation of floral meristems. In Arabidopsis, *AP2* controls the establishment of floral meristem identity in addition to its later role in the specification of floral organ identity (Bowman *et al*., 1993). Under SD conditions, *ap2* mutant flowers showed enhanced inflorescence‐like characteristics (Okamuro *et al*., 1997). We speculate that *AP2‐*like genes might have an ancestral role promoting floral meristem establishment in Angiosperms.

*AP2‐*like genes alone are not sufficient to establish floral meristem identity, however. These genes are expressed in many other tissues (including root, leaf and shoot; Figure 1d) and *AP2* overexpression does not produce ectopic flowers in vegetative tissues. These observations indicate that in order to promote floral meristems, the *AP2* genes require the activation of additional genes involved in the reproductive phase. For example, the Arabidopsis triple mutant *ap1 cal ful* shows a non‐flowering phenotype in which plants continuously generate leafy shoots in place of flowers (Ferrándiz *et al*., 2000). Combined mutations in the homologous wheat genes *vrn1 ful2 ful3* resulted in spikes where the lateral spikelets were replaced by vegetative tillers (Li *et al*., 2019). In rice, the triple mutant of the *SEP‐*like genes *osmads1 osmads5 osmads34* also showed a significant increase in the number of sterile bracts (Wu *et al*., 2018). These results suggest that the expression of A‐class and some early E‐class MADS‐box genes may be a prerequisite for the *AP2‐*like genes to promote the differentiation of axillary floral meristems.

### *AP2L2* and *AP2L5* affect floral organ identity

The *ap2l2* a*p2l5* mutant was still able to produce distal florets that featured various floral organ abnormalities, including an absence of palea, homeotic transformations of lodicules and the adjacent stamen into carpelloid structures, and a reduced number of stamens. Floral abnormalities were also observed in the *ap2l5* mutant, but at a lower frequency (Table 1). The only abnormality detected in the *ap2l2* mutant was an enlargement of the lodicules (Figure S1). To understand better the floral phenotypes observed in the *AP2‐*like mutants, we characterized the transcript levels of several MADS‐box genes known to be involved in the ABCE model, for the determination of floral organ identity (Coen and Meyerowitz, 1991; Theissen *et al*., 2016).

The transcript levels of A‐class genes *VRN1*,* FUL2* and *FUL3*, which control early stages of spike and spikelet development (Li *et al*., 2019), did not significantly differ between *ap2l2* a*p2l5* and the wild type control (Figure 5b). This result suggests that the A‐class MADS‐box genes operate upstream of *AP2L2* and *AP2L5* genes. The B‐class genes *TaPI1* and *TaAP3*, the orthologs of which control lodicule and stamen development in rice and maize (Ambrose *et al*., 2000; Nagasawa *et al*., 2003; Whipple *et al*., 2004; Yao *et al*., 2008), showed a greater than 10‐fold reduction in transcript levels in the *ap2l5* and *ap2l2 ap2l5* mutants relative to the control (Figure 5c). This result may explain the developmental defects observed in the lodicules and stamens in these two mutants. Although no significant differences in transcript levels between *ap2l2* and the wild type were detected for *TaPI1* and *TaAP3*, their transcript levels were consistently lower in *ap2l2 ap2l5* relative to *ap2l5* (not significant), suggesting a limited role of *AP2L2* in the regulation of B‐class genes. This result may explain the increase in lodicule size observed in *ap2l2* (Figure S1d–f) and the decrease in lodicule size in the *rAp2l2* plants (Figure 6m).

Two closely related C‐class *AGAMOUS*‐like genes, *TaAG1* (*OsMADS58*) and *TaAG2* (*OsMADS3*), have been identified in monocots (Yamaguchi *et al*., 2006), and both were highly upregulated in the wheat *ap2l2 ap2l5* mutant (Figure 5d). The rice homologs have partially sub‐functionalized roles in the specification of stamens and carpels (Yamaguchi *et al*., 2006; Dreni *et al*., 2011), so their increased expression in the *ap2l2 ap2l5* wheat mutant may explain the generation of ectopic carpelloid organs replacing the lodicule and adjacent stamen. In Arabidopsis, the negative regulation of *AG* by AP2 and the expansion of the *AG* expression domain in *ap2* mutants is a central concept in the classical ABC model (Bowman *et al*., 1991; Coen and Meyerowitz, 1991; Drews *et al*., 1991). Our data suggest that this interaction also persists in wheat.

The *SEP*‐like genes (E class), are divided into two subfamilies (Malcomber and Kellogg, 2005). *TaSEP4* and *TaSEP3* belong to the *SEP3* subfamily, which controls lodicule, stamen and carpel identity in rice (Cui *et al*., 2010). The other *SEP*‐like genes, including *TaSEP2*,* TaSEP6* and *TaSEP5*, belong to the LOFSEP subfamily and are involved in the specification of most spikelet and floral organs (Cui *et al*., 2010; Wu *et al*., 2018). Two of the LOFSEP genes, *TaSEP5* (*OsMADS34*) and *TaSEP6* (*OsMADS5*) are expressed earlier than the other *SEPALLATA* genes in both rice (Wu *et al*., 2018) and wheat (Figure S4d). In rice, *OsMADS34* (= *PAP2*) is the earliest expressed among the *SEP*‐like genes and regulates the timing of the transition between branches and spikelet meristems (Kobayashi *et al*., 2010). Interestingly, mutations in *ap2l5* and *ap2l5 ap2l2* only affect the expression of the three SEP‐like genes expressed later in flower development (Figure S4e). This result is consistent with the effect of *AP2L2* and *AP2L5* in the regulation of B‐ and C‐class but not A‐class genes, as described above. The 10‐fold downregulation of *TaSEP2* (*OsMADS1*) in *ap2l5* and *ap2l5 ap2l2* may contribute to the conversion of lemmas to glumes, as overexpression of the rice ortholog *OsMADS1* results in the conversion of rudimentary glumes to lemmas (Prasad *et al*., 2001).

Genetic studies in rice and maize have shown that the functions of the B‐, C‐ and E‐class genes are relatively well conserved between eudicots and grasses, but that the role of A‐class genes is less clear (Litt, 2007; Causier *et al*., 2010). Mutations in Arabidopsis *AP2*, an A‐class gene (Theissen *et al*., 2016), affect development of the first (sepal) and second (petal) whorls. In strong *ap2* mutants, the number of organs in the third whorl (stamens) is also reduced, whereas the fourth whorl is normal in all *ap2* mutant alleles (Kunst *et al*., 1989; Drews *et al*., 1991). In the wheat *ap2l2 ap2l5* mutant, lemmas resemble glumes and the development of lodicules and paleas are affected, indicating that these genes also control the identity/development of the perianth. Moreover, the reduced number of stamens in whorl 3 is similar to the phenotype of the strongest *ap2* alleles in Arabidopsis.

The homeotic conversion of lodicules to carpels observed in the *ap2l2 ap2l5* mutants is different from the petal‐to‐stamen conversion observed in Arabidopsis *ap2* mutants. We propose that the floret phenotypes observed in the *ap2l5* and *ap2l2 ap2l5* mutants probably result from a failure to specify floral meristem fate (Litt 2007; Causier *et al*. 2010) combined with the misregulation of B‐class and C‐class genes (Figure 5). In the Arabidopsis *ap2* mutants, the unchanged activity of B‐class genes and the expansion of the *AG* expression domain converts petals into stamens. By contrast, the reduced expression of B‐class genes and increased expression *AG‐*like (C‐class) genes in the wheat *ap2l2 ap2l5* mutant result in the conversion of lodicules into carpelloid organs.

### *AP2L2* affects the swollen area of lodicules

The swelling of the lodicules is necessary to force apart the lemma and palea at anthesis, allowing the stamen filaments to extrude the anthers that release the pollen. There are natural variants of barley where the palea and lemma remain tightly closed throughout the period of pollen release, a character known as cleistogamy (Nair *et al*., 2010; Ning *et al*., 2013). In barley the locus regulating cleistogamy (*Cly1/Zeo*) was mapped to the distal region of chromosome arm 2H (Turuspekov *et al*., 2004). Cloning of this gene revealed that it was a homolog of Arabidopsis *AP2* (Nair *et al*., 2010) that belongs to the same clade as *AP2L2* in wheat (henceforth, *HvAP2L2*; Figure 1). Cleistogamous flowering in barley is caused by a mutation in the binding site of miR172 in *HvAP2L2* (*cly1.b*), which reduces mRNA cleavage (Nair *et al*., 2010) and results in a higher accumulation of HvAP2L2 protein in the lodicules and reduced lodicule size (Anwar *et al*., 2018). An epigenetic modification in a regulatory region has been postulated to explain the reduced expression of *HvAP2L2* and the increased swelling of the lodicules (although still insufficient to open the floret) in barley accession SV235 relative to *cly1.b*, in spite of having the same miR172 target sequence (Wang *et al*., 2015b).

In wheat, the *ap2l2* mutant generated larger lodicules than in wild0type plants (Figure S1). By contrast, wheat lines with the *rAp2l‐A2* and *rAp2l‐B2* alleles developed florets with smaller lodicules in both tetraploid (Figure 6m–n) and hexaploid wheat (Figure S5i). Thus, similarly to barley, the swollen area of the lodicules seems to be inversely correlated with the *AP2L2* levels. The lodicules were always present in the *ap2l2* mutant, but they were missing in 21.6% of *ap2l5* flowers and 100% of *ap2l2 ap2l5* flowers (Table 1). These results suggest that both *AP2L2* and *AP2L5* play critical and redundant roles in lodicule development.

A previous characterization of natural variation in *AP2L2* in 63 wheat accessions found no natural variation in the miR172 binding site within the *AP2L‐A2*,* AP2L‐B2* or *AP2L‐D2* homeologs (Ning *et al*., 2013). Although there is a polymorphism in the second position between a thymine in the A and D genomes and a cytosine in the B genome, these mutations are in the 5′ end of the target site and are predicted to have little to no effect on miRNA activity (Liu *et al*., 2014). Moreover, a similar mutation in the 5′ end of the miR172 target site of *HvAP2L2* from barley Morex does not have phenotypic effects (Nair *et al*., 2010).

We looked at 72 additional wheat accessions comprising two diploid, 11 tetraploid and 59 hexaploid accessions (Table S4), and we failed to detect variation in the miR172 binding site of the different *AP2L2* homeologs. Therefore, the two *rAp2l2* alleles identified in this study in tetraploid and hexaploid wheat represent useful tools to modulate plant height, spike compactness and lodicule function. In wheat, reduced anther extrusion and closed flowering has been associated with a lower risk of *Fusarium* head blight infections (Kubo *et al*., 2010, 2013). It would be interesting to combine *rAp2l‐A2* and *rAp2l‐B2* to see if they are sufficient to induce cleistogamy in polyploid wheat.

### *AP2L5*, but not *AP2L2*, affects spikelet and floret number and heading time

#### AP2L5 increases spikelet number

The *ap2l5* mutants showed a significant reduction in spikelet number per spike (SNS), which indicates a premature transition of the inflorescence meristem to a terminal spikelet. This effect was not observed in the *ap2l2* mutant, and was not enhanced in *ap2l2 ap2l5* relative to *ap2l5* (Figure S2a). Mutations in the miR172 binding site of *AP2L5* resulted in increased SNS, indicating a delay in the transition between IM and the terminal spikelet (Greenwood *et al*., 2017). Similar effects were reported in rice, where *snb osids1* double mutants showed fewer branches (Zhu *et al*., 2009; Lee and An, 2012) whereas MIM172 plants had increased branching (Wang *et al*., 2015a). The tassels of maize *sid1 isd1* mutants also showed reduced branching (Chuck *et al*., 2008), whereas the de‐repression of *AP2‐*like genes in *ts4* and *Ts6* (*rIDS1*) resulted in increased tassel branching (Chuck *et al*., 2007). These results suggest a conserved role of grass *AP2L5/IDS1 SID* orthologs in delaying the transition of inflorescence meristems (or branch meristems) into spikelets.

The previous results seem related to the role of *AP2* in Arabidopsis in maintaining the stem cell niche (= apical initial cells) and the proliferative nature of the shoot meristem (Wurschum *et al*., 2006; Balanza *et al*., 2018). In the Arabidopsis IM, the MADS‐box protein FUL directly and negatively regulates the accumulation of *AP2*, and *ful* mutants produce more fruits than the wild type. (Balanza *et al*., 2018). Interestingly, loss‐of‐function mutations in wheat *FUL2* or *VRN1* (homologs of Arabidopsis FUL) result in significant increases in SNS and number of grains per spike (Li *et al*., 2019), suggesting the potential conservation of a similar regulatory module in wheat and Arabidopsis inflorescence meristems.

#### AP2L5 controls floret number

The *ap2l5* mutant (but not the *ap2l2* mutant) showed a significant increase in floret number per spikelet (Figure 2e). The *ap2l2 ap2l5* mutants also exhibited a large number of organs per spikelet, but in this case, most of them were sterile bracts (Figure 2b,e). In maize and rice, the *ids1 sid1* and *osids1 snb* mutants produced multiple sterile bracts before the development of a terminal spikelet (Chuck *et al*., 2007; Lee and An, 2012). These observations indicate a role of *AP2L5* and its orthologs in reducing the meristematic activity of the spikelet meristem and the number of florets that can be generated. We currently do not know why mutations in the *ap2‐*like genes in grasses operate differently in the IM (reducing the number of lateral organs) than in the SM (increasing the number of lateral organs).

#### AP2L5 delays heading time

If spikelets are generated by the IM at the same rate in different genotypes, a reduction in SNS is expected to accelerate heading time. This was observed in the *ap2l5* mutants, which flowered approximately 4 days earlier than the wild‐type controls (Figure S2); however, Ubi::miR172 plants produce one fewer leaf than the wild type, suggesting that one or more *AP2*‐like genes also affect the transition of the SAM from the vegetative to the reproductive stage. *AP2*‐like genes are known repressors of the flowering promoting gene *FT* in many species, and mutation in several *ap2*‐like genes or overexpression of miR172 produce early flowering (Huijser and Schmid, 2011; Zhu and Helliwell, 2011). The flowering phenotype of wheat *ap2*‐like mutants suggests that *AP2L5*, but not *AP2L2*, may have a conserved role in the regulation of *FT* expression.

In summary, *AP2L5* in wheat seems to have retained a broader role controlling reproductive development (spikelet and floret number and heading time) than *AP2L2*, which seems to be more restricted to spikelet and floret development.

## Conclusion

The results from this and previous studies show that the balance in the expression of miR172 and *AP2‐*like genes is crucial for the correct development of the grass spikelet, and that this balance has been altered during the domestication of wheat and barley. Both the domesticated allele of wheat gene *Q*, a major determinant of the free‐threshing and compact spike character, and the barley *Cly1/Zeo1* gene, which confers compact spike and cleistogamy, resulted from spontaneous mutations in their miR172 target sites that reduce miR172 cleavage activity. These examples show the potential for the modulation of the activity of the *AP2*‐like genes to control important agronomic traits. The two *rAp2l2* alleles identified in this study provide tools to explore the value of the resulting modifications in plant height and spike compactness in different wheat classes and/or in different environments.

In addition to its potential practical applications, this study provided insights on the critical and redundant roles of *AP2L2* and *AP2L5* in the development of axillary floral meristems and the differentiation of lemma characteristics in the subtending bract. Finally, our study showed an essential role of these genes in the development of lodicules and on the regulation of B‐, C‐ and late E‐class MADS‐box floral genes.

## Experimental Procedures

### Plant materials and growth conditions

The tetraploid wheat variety Kronos used in this study has a spring growth habit determined by the *Vrn‐A1c* allele. Kronos also has the *Q* allele, which confers the subcompact spike phenotype and the free‐threshing character. TILLING populations of Kronos mutagenized with ethyl methane sulphonate (EMS) (Uauy *et al*., 2009) were used to screen for mutants of the *AP2L2* gene. The two selected truncation mutations and the mutation in the miR172 target site were confirmed in M_4_ grain using the genome‐specific primers described in Table S5.

The effect of the mutations on the *AP2L2* transcripts was verified by RT‐PCR on RNA extracted from leaves of the *ap2l2* mutant. The genome‐specific primers are described in Table S5. For the K3634 mutation we also tested nested PCR, but we were unable to detect the transcript in the mutant. For all experiments, grains were first cold imbibed for 2–4 days at 4°C. The plants were grown in cones in PGR15 growth chambers (Conviron, http://www.conviron.com) adjusted to 16 h of light (22°C) and 8 h of darkness (18°C). The intensity of the sodium halide lights measured at the height of plant heads was (~260 μm m^−2^ s^−1^). The line with the mutation in the miR172 target site of the *AP2L*‐*B2* mutant was obtained in the winter hexaploid variety Wedgetail, which was mutagenized using sodium azide (Chandler and Harding, 2013). Primers used to genotype the mutant line are listed in Table S5. Phenotyping for co‐segregation analysis was performed in a glasshouse with 16 h of light (22°C) and 8 h of dark (18°C), after 7 weeks of vernalization.

### qRT‐PCR

RNA samples were extracted using the Spectrum Plant Total RNA Kit (Sigma‐Aldrich, https://www.sigmaaldrich.com). We followed Protocol A that allows for the purification of total RNA including small RNA molecules. Total RNA was treated with RQ1 RNase‐free DNase (Promega, https://www.promega.com). cDNA synthesis was carried out using SuperScript II Reverse Transcriptase (Invitrogen, now ThermoFisher Scientific, https://www.thermofisher.com). mRNAs were reverse transcribed starting from 1 μg of total RNA and using OligodTv primer. The product from the first‐strand synthesis was diluted 1 in 20, and 5 μl of diluted cDNAs was used in the qRT‐PCR reaction, which was performed using SYBR Green and a 7500 Fast Real‐Time PCR system (Apply Biosystems, a brand of ThermoFisher Scientific). The *ACTIN* gene was used as an endogenous control for mRNAs. Primers are listed in Table S5.

### Morphological traits

To study the anatomical changes in the glumes and lemmas of the different genotypes we made transverse sections of dry glumes and lemmas of fully developed spikes. We boiled the organs in water and then sectioned them by hand using a razor blade. Transverse sections were stained with toluidine blue O solution for 30 s. Images of the stained sections and dissected floret organs were digitally captured using a stereo‐dissecting scope.

### Scanning electron microscopy (SEM)

Spikes at different developmental stages were dissected, fixed for a minimum of 24 h in FAA (50% ethanol, 5% v/v acetic acid, 3.7% v/v formaldehyde), rinsed twice in the same buffer, and dehydrated through a graded ethanol series to absolute ethanol. Samples were critical‐point dried in liquid CO_2_ (tousimis^®^ 931 series critical point drier; tousimis, https://tousimis.com), mounted on aluminum stubs, coated with gold (Denton Desk II Sputter Coater) and examined with a ThermoFisher Quattro scanning electron microscope operating at 5 kV. Images were recorded with a slow scan for high definition and saved as TIFF files.

### Phylogenetic analysis

The complete protein sequences of the different *AP2*‐like genes from Brachypodium, maize, rice and Arabidopsis were obtained from the Phytozome web resource (https://phytozome.jgi.doe.gov/pz/portal.html). Sequences from barley were obtained from the International Barley Sequencing Consortium (https://webblast.ipk-gatersleben.de/barley_ibsc/viroblast.php). Protein sequences from wheat were obtained from Wheat Genome RefSeqv 1.1. Evolutionary analysis was conducted in mega x. For analysis, we used a region that included the two AP2 domains.

### Wheat transformation

Transgenic wheat plants were generated at the UC Davis Plant Transformation Facility (http://ucdptf.ucdavis.edu/) using the Japan Tobacco (JT) technology licensed to UC Davis. Immature embryos from Kronos were transformed using *Agrobacterium EHA105*. The selection of transgenic plants was conducted using hygromycin, and transgene insertion was validated by DNA extraction and PCR.

## Conflict of Interest

The authors of this manuscript declare that they do not have any conflicts of interest.

## Author Contributions

JMD and JD conceived the study. JMD performed most of the experimental work. JMD and JD analysed the data. JJ was responsible for the scanning electron microscopy studies. JRG and EJF. identified and characterized the hexaploid wheat variety Wedgetail with an induced mutation in the miR172 target site of *AP2L2*. JMD wrote the first draft of the manuscript and all coauthors contributed ideas and corrections to the subsequent versions.

## Data Statement

This article does not include large data sets, but all the data and genetic materials are available from the authors upon request.

## Supporting information


**Figure S1.** *AP2L2* induced mutants.
**Figure S2.** Spikelet number and heading time for the wild type and *ap2l2*,* ap2l5* and *ap2l2* *ap2l5* mutants.
**Figure S3.** Scanning electron microscopy images of dissected apices from the wild type (Wt) and the *ap2l2*,* ap2l5* and *ap2l2* *ap2l5* mutants.
**Figure S4**. Transcript levels of wheat MADS‐box genes involved in floral organ identity during spike development.
**Figure S5.** Mutation in the miR172 binding site of *AP2L‐B2* in hexaploid wheat (*rAp2l‐B2*).

**Table S1**. Locus name for the different wheat *AP2*‐like genes.
**Table S2.** Reciprocal BLASTN searches for wheat homologs of *SNB/SID1*.
**Table S3**. Wheat orthologs of MIKC‐type MADS‐box genes involved in the ABCE flowering model.
**Table S4**. Natural variation in miR172 target site of *AP2L2*.
**Table S5**. Primers used in this study.
