# Supplementary material for: APETALA 2‐like genes AP2L2 and Q specify lemma identity and axillary floral meristem development in wheat
Source: Plant J. 2019 Oct 15;101(1):171–87. doi: 10.1111/tpj.14528 (PMC6972666; doi:10.1111/tpj.14528)
Supplement: Supplementary file 1 — Figure S1. AP2L2 induced mutants. Figure S2. Spikelet number and heading time for the wild type and ap2l2, ap2l5 and ap2l2 ap2l5 mutants. Figure S3. Scanning electron microscopy images of dissected apices from the wild type (Wt) and the ap2l2, ap2l5 and ap2l2 ap2l5 mutants. Figure S4. Transcript levels of wheat MADS‐box genes involved in floral organ identity during spike development. Figure S5. Mutation in the miR172 binding site of AP2L‐B2 in hexaploid wheat (rAp2l‐B2). [file TPJ-101-171-s001.docx]

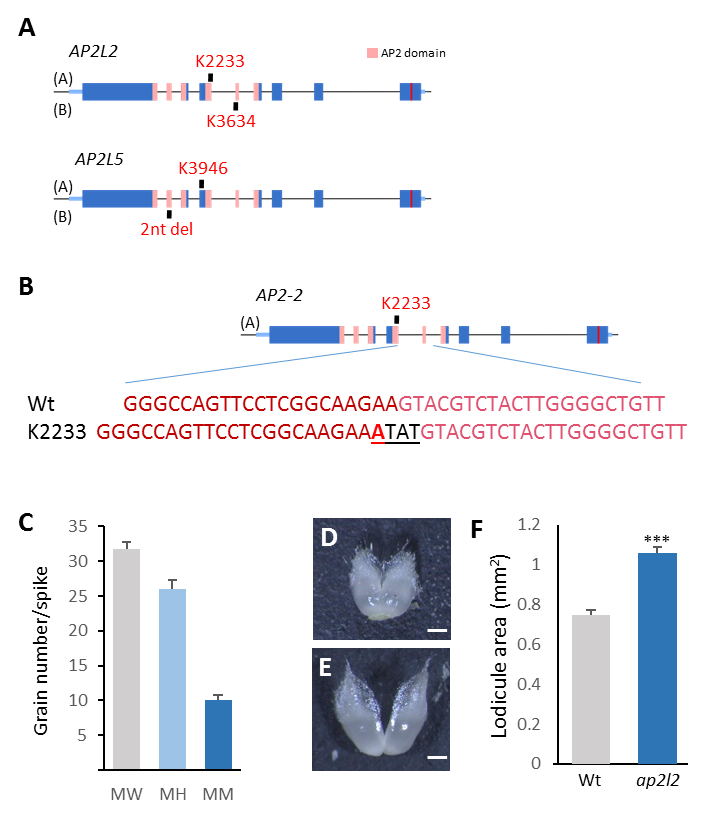


**Figure S1**. ***AP2L2* induced mutants.** **(A)** Genomic structure of *AP2L2* and *AP2L5* genes showing the position of the Kronos mutations K2233, K3634 and K3946. Exons are in blue, AP2 domains in pink, and the miR172 target site in red. Mutations in the A homeolog are shown above the gene structure and in the B homeolog below. The *AP2L-5B* homeolog has a natural 2 nt deletion. **(B)** Effect of the K2233 mutation in the splicing of *AP2L-2A* homeolog. The correct (wt) and mutant (K2233) splicing of the 4^th^ and 5^th^ exons are shown below the genomic structure. The K2233 line has a G-to-A change (red) in the splicing donor site of the 4^th^ intron. That mutation results in the use of a nearby GT site and retention of 4 extra intronic nucleotides (underlined). **(C)** Grain number per spike in K3634 homozygous mutant segregating for the K2233 mutation. MW= K3634 mutant/ K2233 wild type; MH= K3634 mutant/ K2233 heterozygous; MM= K3634 mutant/ K2233 mutant. Note the reduced number of grains in the double *ap2l-2A* *ap2l-2B* mutant (MM). **(D-E)** Dissected lodicules from wild type **(D)** and *ap2l2* **(E)** florets, scale bar = 0.5mm. **(F)** Swollen area in lodicules from wild type (Wt) and *ap2l2* florets. Bars represent mean ± s.e.m. (based on 20 lodicules from 10 florets). *** indicates statistically significant differences (*P* < 0.001) by *t*-test.


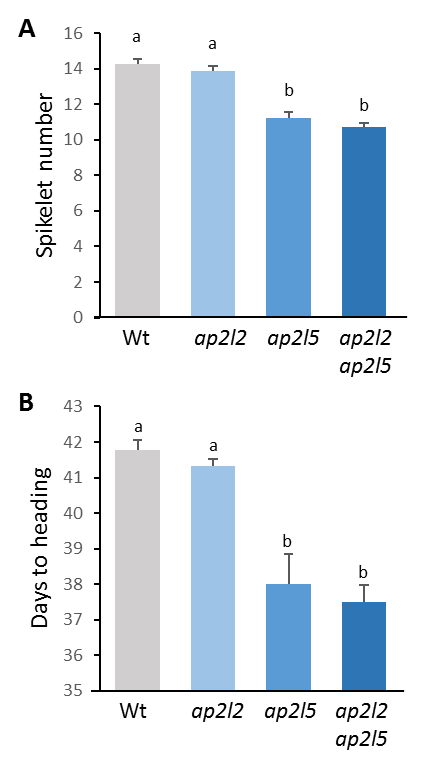


**Figure S2.** **Spikelet number and heading time for wild type and *ap2l2*, *ap2l5* and *ap2l2* *ap2l5* mutants.** **(A)** Spikelet number in the primary spike and **(B)** days to heading for wild type Kronos (Wt), *ap2l2*, *ap2l5* and *ap2l2* *ap2l5* mutants. Bars represent mean ± s.e.m. and different letters above the bars indicate statistically significant differences (P<0.05) by Student–Newman–Keuls test.


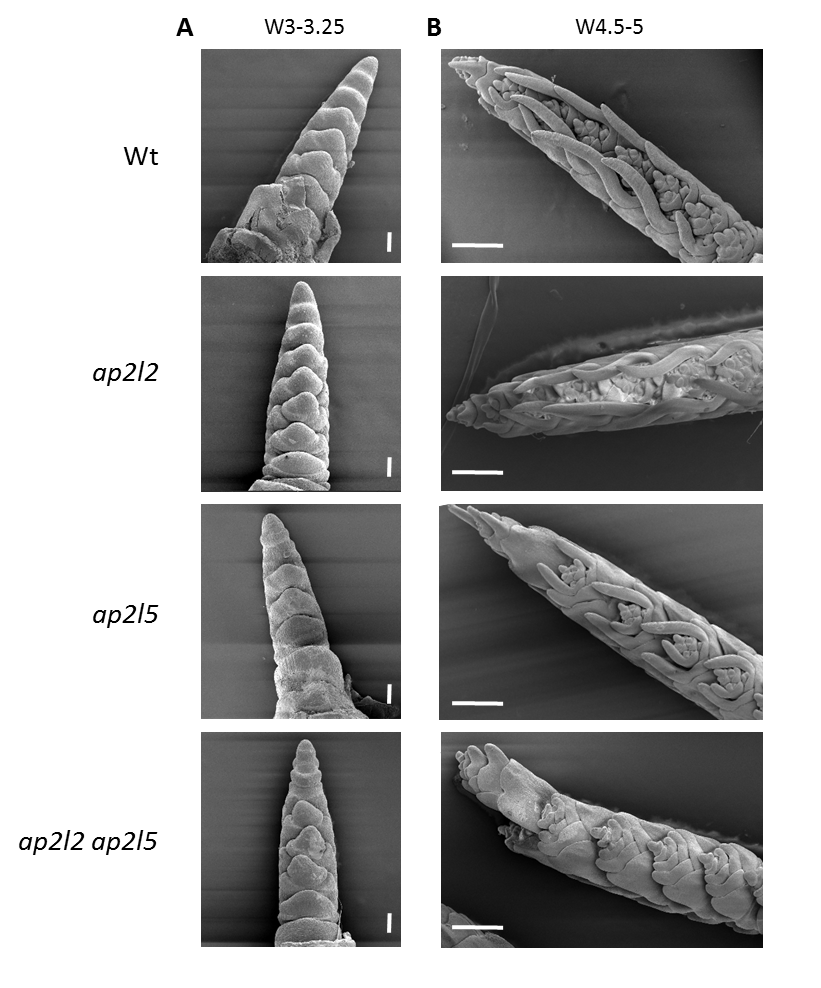


**Figure S3**. **Scanning electron microscopy images of dissected apices from wild type (Wt) and *ap2l2*, *ap2l5* and *ap2l2* *ap2l5* mutants.** **(A)** Spike development at Waddington stage W3-3.25. Scale bars are 100 µm **(B)** Spike development at Waddington stage W4.5-5 stages. Scale bars are 500 µm.


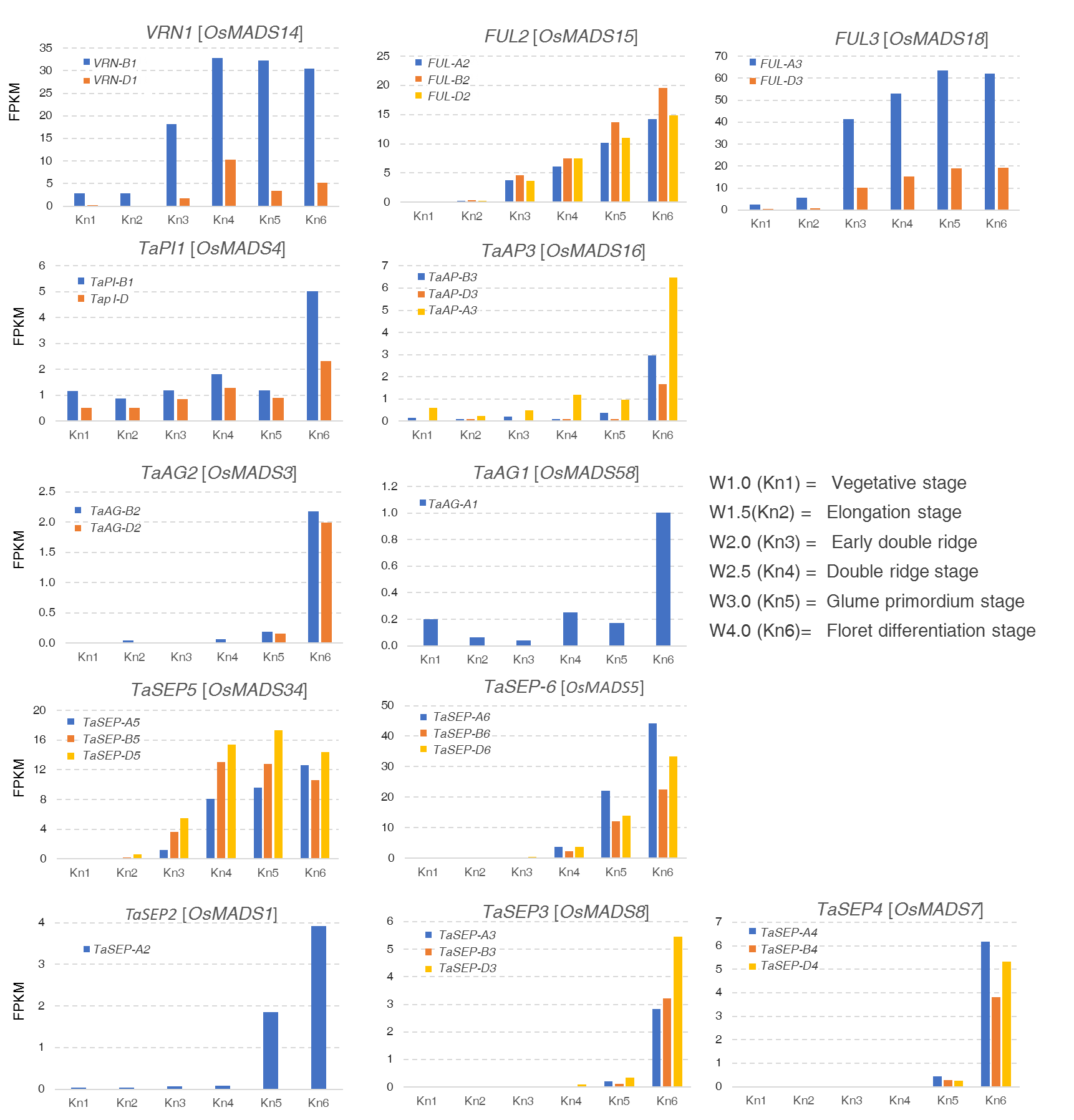


**E**

**D**

**C**

**B**

**A**

**Figure S4**. **Transcript levels of wheat MADS-box genes involved in floral organ identity during spike development.** Based on previously published RNASeq dataset (Li et al., 2018, Scientific Reports 8:15338). **(A)** Class A genes: *VRN1*, *FUL2* and *FUL3*. **(B)** Class B genes: *TaPI1*and *TaAP3*. **(C)** Class C genes: *TaAG2* and *TaAG1*. **(D)** Class E genes expressed earlier in spikelet development: *TaSEP5* (*= PAP2*) and *TaSEP6*. **(E)** Class E genes expressed later in spikelet development: *TaSEP2*, *TaSEP8* and *TaSEP7*. FPKM= Fragments per kb of transcript per million mapped reads. W1.0 = vegetative stage, W1.5 = elongating apex, W2.0 = early double ridge stage, W2.5 = double ridge stage, W3.0 = glume primordium differentiation stage, W4.0 = floret primordium differentiation stage.


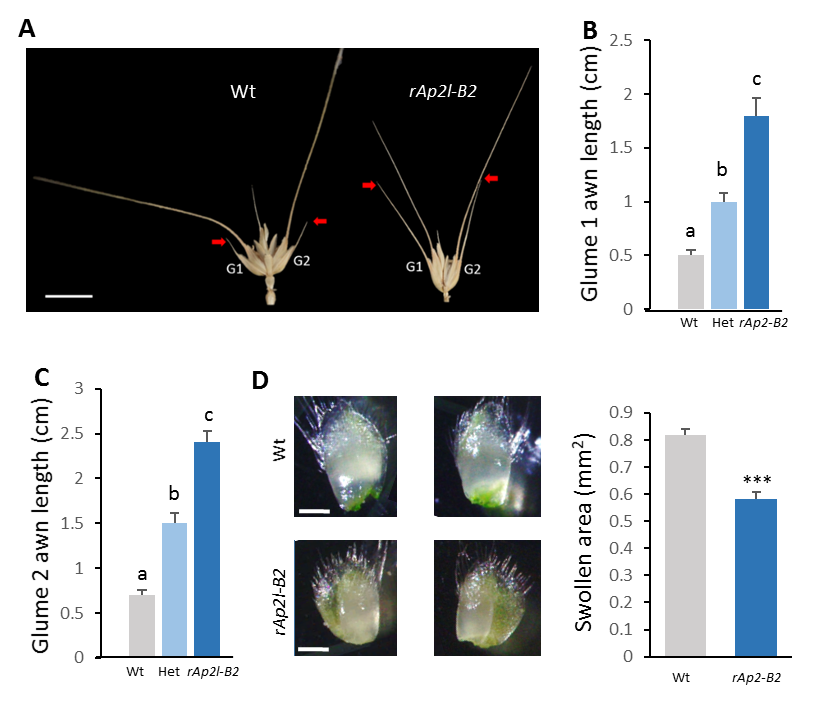


**Figure S5.** **Mutation in the miR172 binding site of *AP2L-B2* in hexaploid wheat (*rAp2l-B2*).** **(A)** Picture showing a representative penultimate spikelet from a wild type (WT) spike and a homozygous mutant (Mut). Red arrows point to glume 1 (G1) and glume 2 (G2) awn tips, scale bar= 1cm. **(B-C)** Length of the awn of the first **(B)** and second **(C)** glumes in the penultimate spikelet of F_2_ plants segregating for *rAp2l-2B* mutation (Wt = homozygous wild type, Het = heterozygous, and Mut = homozygous mutant plants) (n ≥ 10). Different letters above the bars indicate statistically significant differences (*P* < 0.05) by Student–Newman–Keuls test. **(D)** Picture of lodicule (left) and graph of average lodicule swollen area (right) from F_2_ plants segregating for the *rAp2l-2B* mutation (Wt = homozygous wild type, and Mut = homozygous *rAp2l-2B*) (n≥16). *** = *P* < 0.0001. In all graphs bars represent ± s.e.m.
