# Supplementary material for: APETALA 2‐like genes AP2L2 and Q specify lemma identity and axillary floral meristem development in wheat
Source: Plant J. 2019 Oct 15;101(1):171–87. doi: 10.1111/tpj.14528 (PMC6972666; doi:10.1111/tpj.14528)
Supplement: Supplementary file 2 — Table S1. Locus name for the different wheat AP2‐like genes. Table S2. Reciprocal BLASTN searches for wheat homologs of SNB/SID1. Table S3. Wheat orthologs of MIKC‐type MADS‐box genes involved in the ABCE flowering model. Table S4. Natural variation in miR172 target site of AP2L2. Table S5. Primers used in this study. [file TPJ-101-171-s002.docx]

**Table S1**. **Locus name for the different wheat *AP2*-like genes**. Wheat homeologs harboring a miR172 target site in the Wheat Genome RefSeqv1.1 and their closest barley, *Brachypodium distachyon* and rice orthologs.

| **Genome** | ***AP2L1*** | ***AP2L2*** | ***AP2L5*** | ***AP2L7*** |
| --- | --- | --- | --- | --- |
| **A** | *TraesCS1A02G058400* | *TraesCS2A02G514200* | *TraesCS5A02G473800* | *TraesCS7A02G744600* |
| **B** | *TraesCS1B02G076300* | *TraesCS2B02G542400* | *TraesCS5B02G486900* | *TraesCS7B02G440400* |
| **D** | *TraesCS1D02G059200* | *TraesCS2D02G515800* | *TraesCS5D02G486600* | *TraesCS7D02G512600* |
| ***Barley*** | *HORVU1Hr1G011800* | *HORVU2Hr1G113880* | *HORVU5Hr1G112440* | *HORVU7Hr1G116220* |
| ***Brachy.*** | *Bradi2g37800* | *Bradi5g24100* | *Bradi1g03880* | *Bradi1g30337* |
| ***Rice*** | *Os05g03040* | *OsSHAT1* | *OsIDS1* | *Os06g43220* |

**Table S2. Reciprocal BLASTN searches for wheat homologs of *SNB/SID1****.* The *Brachypodium* gene *Bradi1g53650* was used as query*.* Note that in rice and *B. stacei* the reciprocal BLASTN returns the correct *Bradi1g53650.* Whereas the closest homologs in *T. urartu* (progenitor of genome A in polyploid wheat), einkorn wheat (genome A^m^), *A. tauschii* (progenitor of genome D in hexaploid wheat), wild emmer Zavitan, rye, and barley, return the *Brachypodium* orthologs of *AP2L5* (blue), *AP2L1* (green), *AP2L7* (orange), and *AP2L2* (red). AP2-like genes of the SNB/SID1 clade are highlighted in gray.

| Query | Species | Blast hit | E-value | Best hit in Wheat | Best reciprocal hit in *B. distachyon* |
| --- | --- | --- | --- | --- | --- |
|  |  |  |  |  |  |
| *Bradi1g53650 (SID1/SNB)* | *O. sativa* | LOC_Os07g13170 (SNB) | 0 | *TraesCS****5****D02G486600* | *Bradi1g53650* |
|  | *B. stacei* | Brast06G171200.1 | 0 | *TraesCS****5****D02G486600* | *Bradi1g53650* |
|  | *T. urartu* | UCW_Tu-k41_contig_3850 | 2.00E-161 | *TraesCS****5****A02G473800* | *Bradi1g03880* |
|  |  | UCW_Tu-k21_contig_2135 | 2.00E-161 | *TraesCS****5****A02G473800* | *Bradi1g03880* |
|  |  | UCW_Tu-k55_contig_4711 | 2.00E-161 | *TraesCS****5****A02G473800* | *Bradi1g03880* |
|  |  | UCW_Tu-k61_contig_1259;tu-k51_contig_1343 | 3.00E-101 | *TraesCS****1****A02G058400* | *Bradi2g37800* |
|  |  | TmoDV92v1_058611 | 4.00E-160 | *TraesCS****5****A02G473800* | *Bradi1g03880* |
|  |  | TmoDV92v1_058610 | 4.00E-160 | *TraesCS****5****A02G473800* | *Bradi1g03880* |
|  |  | TmoDV92v1_058604 | 4.00E-160 | *TraesCS****5****A02G473800* | *Bradi1g03880* |
|  |  | TmoDV92v1_058605 | 3.00E-156 | *TraesCS****5****A02G473800* | *Bradi1g03880* |
|  | *T. monococcum* DV92 | TmoDV92v1_058609 | 8.00E-125 | *TraesCS****5****A02G473800* | *Bradi1g03880* |
|  |  | TmoDV92v1_058606 | 1.00E-122 | *TraesCS****5****A02G473800* | *Bradi1g03880* |
|  |  | TmoDV92v1_058608 | 4.00E-110 | *TraesCS****5****A02G473800* | *Bradi1g03880* |
|  |  | TmoDV92v1_058607 | 4.00E-110 | *TraesCS****5****A02G473800* | *Bradi1g03880* |
|  |  | TmoDV92v1_013631 | 6.00E-101 | *TraesCS****1****A02G058400* | *Bradi2g37800* |
|  |  | TmoDV92v1_013629 | 6.00E-101 | *TraesCS****1****A02G058400* | *Bradi2g37800* |
|  | *A. tauschii* | lcl\|5342.1 | 2.00E-25 | *TraesCS****5****D02G486600* | *Bradi1g03880* |
|  |  | gnl\|TRIDC1\|WEWZ_5B | 5.00E-26 | *TraesCS****5****B02G486900* | *Bradi1g03880* |
|  |  | gnl\|TRIDC1\|WEWZ_5A | 5.00E-26 | *TraesCS****5****A02G473800* | *Bradi1g03880* |
|  |  | gnl\|TRIDC1\|WEWZ_1B | 2.00E-12 | *TraesCS****1****B02G076300* | *Bradi2g37800* |
|  | Wild emmer Zavitan | gnl\|TRIDC1\|WEWZ_1A | 2.00E-12 | *TraesCS****1****A02G058400* | *Bradi2g37800* |
|  |  | gnl\|TRIDC1\|WEWZ_7B | 4.00E-11 | *TraesCS****7****B02G440400* | *Bradi1g30337* |
|  |  | gnl\|TRIDC1\|WEWZ_7A | 2.00E-10 | *TraesCS****7****A02G744600* | *Bradi1g30337* |
|  |  | gnl\|TRIDC1\|WEWZ_2B | 6.00E-10 | *TraesCS2B02G542400* | *Bradi5g24100* |
|  |  | gnl\|TRIDC1\|WEWZ_2A | 4.00E-08 | *TraesCS2A02G514200* | *Bradi5g24100* |
|  | *S. cereale* (Rye) | gnl\|Scv2CTG\|Lo7_v2_contig_90 | 8.00E-27 | *TraesCS****5****D02G486600* | *Bradi1g03880* |
|  |  | gnl\|Scv2CTG\|Lo7_v2_contig_144818 | 3.00E-11 | *TraesCS****7****A02G744600* | *Bradi1g30337* |
|  |  | gnl\|Scv2CTG\|Lo7_v2_contig_2885418 | 2.00E-08 | *TraesCS****1****B02G076300* | *Bradi2g37800* |
|  |  | gnl\|Scv2CTG\|Lo7_v2_contig_3792 | 2.00E-08 | *TraesCS2D02G515800* | *Bradi5g24100* |
|  |  | gnl\|Scv2CTG\|Lo7_v2_contig_244 | 4.00E-04 | *TraesCS****5****A02G473800* | *Bradi1g03880* |
|  |  | gnl\|Scv2CTG\|Lo7_v2_contig_1344667 | 0.35 | *TraesCS****1****B02G076300* | *Bradi2g37800* |
|  | *H. vulgare* | HORVU5Hr1G112440.1 | 2.00E-160 | *TraesCS5A02G473800* | *Bradi1g03880* |
|  |  | HORVU1Hr1G011800.24 | 4.00E-94 | *TraesCS1D02G059200* | *Bradi2g37800* |
|  |  | HORVU2Hr1G113880.23 | 7.00E-91 | *TraesCS2D02G515800* | *Bradi5g24100* |
|  |  | HORVU7Hr1G116220.9 | 1.00E-49 | *TraesCS7B02G440400* | *Bradi1g30337* |

* The *SID1/SNB* sequence from *Brachypodium distachyon* (*Bradi1g53650*) was used in a BLASTN search in genomic and transcriptomic sequencing data from:

*O. sativa*, *Brachypodium stacei* (<https://phytozome.jgi.doe.gov/pz/portal.html>).

*T. urartu* (<https://wheat.pw.usda.gov/GG2/WheatTranscriptome/viroblast/viroblast.php>).

*T. monococcum* DV92 (<https://dubcovskylab.ucdavis.edu/wheat-expression-database>).

*A. tuaschii* (<http://aegilops.wheat.ucdavis.edu/ATGSP/blast.php>).

Wild emmer Zavitan (<https://wheat.pw.usda.gov/GG3/wildemmer_blast>).

*S. cereal* (RYE) (<https://wheat.pw.usda.gov/cgi-bin/seqserve/blast_rye.cgi>).

*H. vulgare* (barley) (<https://webblast.ipk-gatersleben.de/barley_ibsc/>).

The BLAST hits obtained were then used to do a reciprocal BLASTN search in *B. distachyon* genomic databases.

The best blast hit in each species are highlighted in yellow.

**Table S3**. Wheat orthologs of MIKC-type MADS-box genes involved in the ABCE flowering model.

|  | **Rice** | |  | **Wheat RefSeq v1.1** | |  | **Published** | |
| --- | --- | --- | --- | --- | --- | --- | --- | --- |
|  | **Name** | **Locus** |  | **A genome** | **B genome** |  | **wheat names** | |
| **B-class** | *OsMADS4* | *LOC_Os05g34940* |  | *TraesCS1A02G264300* | *TraesCS1B02G275000* |  | *TaPI1^a^* | WPI-1^b^ |
|  | *OsMADS16* | *LOC_Os06g49840* |  | *TraesCS7A02G383800* | *TraesCS7B02G286600* |  | *TaAP3^a^* | WAP3^b^ |
| **C-class** | *OsMADS3* | *LOC_Os01g10504* |  | *TraesCS3A02G314300* | *TraesCS3B02G157500* |  | *TaAG2^a^* | WAG-2^c^ |
|  | *OsMADS58* | *LOC_Os05g11414* |  | *TraesCS1A02G125800* | *TraesCS1B02G144800* |  | *TaAG1^a^* | WAG-1^c^ |
| **E-class** | *OsMADS1* | *LOC_Os03g11614* |  | *TraesCS4A02G058900* | *TraesCS4B02G245700* |  | *TaSEP2^a^* | *WLHS1^d^* |
|  | *OsMADS34* | *LOC_Os03g54170* |  | *TraesCS5A02G391800* | *TraesCS5B02G396700* |  | *TaSEP5^a^* |  |
|  | *OsMADS5* | *LOC_Os06g06750* |  | *TraesCS7A02G122000* | *TraesCS7B02G020800* |  | *TaSEP6^a^* |  |
|  | *OsMADS7* | *LOC_Os08g41950* |  | *TraesCS7A02G260600* | *TraesCS7B02G158600* |  | *TaSEP4^a^* | *WSEP ^d^* |
|  | *OsMADS8* | *LOC_Os09g32948* |  | *TraesCS5A02G286800* | *TraesCS5B02G286100* |  | *TaSEP3^a^* |  |

^a^ Paolacci AR, Tanzarella OA, Porceddu E, Varotto S, Ciaffi M (2007). Molecular and phylogenetic analysis of MADS-box genes of MIKC type and chromosome location of SEP-like gene in wheat (Triticum aestivum L.). Mol Genet Genomics 278:689–708.

^b^ Hama E, Takumi S, Ogihara Y, Murai K (2004). Pistillody is caused by alterations to the class-B MADS-box gene expression pattern in alloplasmic wheats. Planta 218:712–720

^c^ Hirabayashi C., Murai K. (2009). Class C MADS-box gene AGAMOUS was duplicated in the wheat genome. Wheat Inf. Serv. 107 13–16.

^d^ Shitsukawa N, Tahira C, Kassai K-I, Hirabayashi C, Shimizu T, Takumi S, Mochida K, Kwaura K, Ogihara Y, Murai K (2007b). Genetic and epigenetic alteration among three homoeologous genes of a class E MADS box gene in hexaploid wheat. Plant Cell 19:1723–1737.

**Table S4**. Natural variation in miR172 target site of *AP2L2*.

| Accessions | miR172 target sequence in *AP2L2* homeologs | | |
| --- | --- | --- | --- |
|  | A | B | D |
| Diploids | | | |
| DV92 | CTGCAGCATCATCACGATTCC |  |  |
| G3116 | CTGCAGCATCATCACGATTCC |  |  |
| Tetraploids | | | |
| 280-1-Yr15 | CTGCAGCATCATCACGATTCC | CCGCAGCATCATCACGATTCC |  |
| D447-DW1 | CTGCAGCATCATCACGATTCC | CCGCAGCATCATCACGATTCC |  |
| Excalibur | CTGCAGCATCATCACGATTCC | CCGCAGCATCATCACGATTCC |  |
| Gredho | CTGCAGCATCATCACGATTCC | CCGCAGCATCATCACGATTCC |  |
| Kronos | CTGCAGCATCATCACGATTCC | CCGCAGCATCATCACGATTCC |  |
| Langdon | CTGCAGCATCATCACGATTCC | CCGCAGCATCATCACGATTCC |  |
| PI 347731 | CTGCAGCATCATCACGATTCC | CCGCAGCATCATCACGATTCC |  |
| PI 503555 | CTGCAGCATCATCACGATTCC | CCGCAGCATCATCACGATTCC |  |
| Rusty | CTGCAGCATCATCACGATTCC | CCGCAGCATCATCACGATTCC |  |
| Svevo | CTGCAGCATCATCACGATTCC | CCGCAGCATCATCACGATTCC |  |
| Zavitan | CTGCAGCATCATCACGATTCC | CCGCAGCATCATCACGATTCC |  |
| Hexaploids | | | |
| 2045A | CTGCAGCATCATCACGATTCC | CCGCAGCATCATCACGATTCC | CTGCAGCATCATCACGATTCC |
| AvocetS | CTGCAGCATCATCACGATTCC | CCGCAGCATCATCACGATTCC | CTGCAGCATCATCACGATTCC |
| Bakahtawar94 | CTGCAGCATCATCACGATTCC | CCGCAGCATCATCACGATTCC | CTGCAGCATCATCACGATTCC |
| Berkut | CTGCAGCATCATCACGATTCC | CCGCAGCATCATCACGATTCC | CTGCAGCATCATCACGATTCC |
| CAP2 | CTGCAGCATCATCACGATTCC | CCGCAGCATCATCACGATTCC | CTGCAGCATCATCACGATTCC |
| Cltr 7635 (LR3) | CTGCAGCATCATCACGATTCC | CCGCAGCATCATCACGATTCC | CTGCAGCATCATCACGATTCC |
| Dharwar_Dry | CTGCAGCATCATCACGATTCC | CCGCAGCATCATCACGATTCC | CTGCAGCATCATCACGATTCC |
| Hahn-RW | CTGCAGCATCATCACGATTCC | CCGCAGCATCATCACGATTCC | CTGCAGCATCATCACGATTCC |
| Inayama | CTGCAGCATCATCACGATTCC | CCGCAGCATCATCACGATTCC | CTGCAGCATCATCACGATTCC |
| UC1036 | CTGCAGCATCATCACGATTCC | CCGCAGCATCATCACGATTCC | CTGCAGCATCATCACGATTCC |
| Klein Chaja | CTGCAGCATCATCACGATTCC | CCGCAGCATCATCACGATTCC | CTGCAGCATCATCACGATTCC |
| Klain Proteo | CTGCAGCATCATCACGATTCC | CCGCAGCATCATCACGATTCC | CTGCAGCATCATCACGATTCC |
| Lalbahadur | CTGCAGCATCATCACGATTCC | CCGCAGCATCATCACGATTCC | CTGCAGCATCATCACGATTCC |
| PBW343 | CTGCAGCATCATCACGATTCC | CCGCAGCATCATCACGATTCC | CTGCAGCATCATCACGATTCC |
| PI 70613 (LR23) | CTGCAGCATCATCACGATTCC | CTGCAGCATCATCACGATTCC | CTGCAGCATCATCACGATTCC |
| PI 519805 | CTGCAGCATCATCACGATTCC | CCGCAGCATCATCACGATTCC | CTGCAGCATCATCACGATTCC |
| UC1419 | CTGCAGCATCATCACGATTCC | CCGCAGCATCATCACGATTCC | CTGCAGCATCATCACGATTCC |
| Pavon | CTGCAGCATCATCACGATTCC | CCGCAGCATCATCACGATTCC | CTGCAGCATCATCACGATTCC |
| RAC875 | CTGCAGCATCATCACGATTCC | CCGCAGCATCATCACGATTCC | CTGCAGCATCATCACGATTCC |
| RIL143 | CTGCAGCATCATCACGATTCC | CCGCAGCATCATCACGATTCC | CTGCAGCATCATCACGATTCC |
| RSI5 | CTGCAGCATCATCACGATTCC | CCGCAGCATCATCACGATTCC | CTGCAGCATCATCACGATTCC |
| 16REG01643 | CTGCAGCATCATCACGATTCC | CCGCAGCATCATCACGATTCC | CTGCAGCATCATCACGATTCC |
| 16REG01644 | CTGCAGCATCATCACGATTCC | CCGCAGCATCATCACGATTCC | CTGCAGCATCATCACGATTCC |
| 2045A | CTGCAGCATCATCACGATTCC | CCGCAGCATCATCACGATTCC | CTGCAGCATCATCACGATTCC |
| Altamo | CTGCAGCATCATCACGATTCC | CCGCAGCATCATCACGATTCC | CTGCAGCATCATCACGATTCC |
| Billings | CTGCAGCATCATCACGATTCC | CCGCAGCATCATCACGATTCC | CTGCAGCATCATCACGATTCC |
| CCW3A37 | CTGCAGCATCATCACGATTCC | CCGCAGCATCATCACGATTCC | CTGCAGCATCATCACGATTCC |
| CCW3A49 | CTGCAGCATCATCACGATTCC | CCGCAGCATCATCACGATTCC | CTGCAGCATCATCACGATTCC |
| Chateau | CTGCAGCATCATCACGATTCC | CTGCAGCATCATCACGATTCC | CTGCAGCATCATCACGATTCC |
| CO960293 | CTGCAGCATCATCACGATTCC | CTGCAGCATCATCACGATTCC | CTGCAGCATCATCACGATTCC |
| Cheyenne | CTGCAGCATCATCACGATTCC | CCGCAGCATCATCACGATTCC | CTGCAGCATCATCACGATTCC |
| Dayn | CTGCAGCATCATCACGATTCC | CCGCAGCATCATCACGATTCC | CTGCAGCATCATCACGATTCC |
| Duster | CTGCAGCATCATCACGATTCC | CCGCAGCATCATCACGATTCC | CTGCAGCATCATCACGATTCC |
| Hank | CTGCAGCATCATCACGATTCC | CCGCAGCATCATCACGATTCC | CTGCAGCATCATCACGATTCC |
| IDO444 | CTGCAGCATCATCACGATTCC | CCGCAGCATCATCACGATTCC | CTGCAGCATCATCACGATTCC |
| KS05HW14-3 | CTGCAGCATCATCACGATTCC | CCGCAGCATCATCACGATTCC | CTGCAGCATCATCACGATTCC |
| LA95135 | CTGCAGCATCATCACGATTCC | CCGCAGCATCATCACGATTCC | CTGCAGCATCATCACGATTCC |
| LCD-STAR | CTGCAGCATCATCACGATTCC | CCGCAGCATCATCACGATTCC | CTGCAGCATCATCACGATTCC |
| Lyman | CTGCAGCATCATCACGATTCC | CCGCAGCATCATCACGATTCC | CTGCAGCATCATCACGATTCC |
| MN98550-5 | CTGCAGCATCATCACGATTCC | CTGCAGCATCATCACGATTCC | CTGCAGCATCATCACGATTCC |
| MN99394-1 | CTGCAGCATCATCACGATTCC | CCGCAGCATCATCACGATTCC | CTGCAGCATCATCACGATTCC |
| McNeal | CTGCAGCATCATCACGATTCC | CCGCAGCATCATCACGATTCC | CTGCAGCATCATCACGATTCC |
| Opata | CTGCAGCATCATCACGATTCC | CCGCAGCATCATCACGATTCC | CTGCAGCATCATCACGATTCC |
| Overley | CTGCAGCATCATCACGATTCC | CCGCAGCATCATCACGATTCC | CTGCAGCATCATCACGATTCC |
| Patwin515 | CTGCAGCATCATCACGATTCC | CCGCAGCATCATCACGATTCC | CTGCAGCATCATCACGATTCC |
| PIONNER26R61 | CTGCAGCATCATCACGATTCC | CTGCAGCATCATCACGATTCC | CTGCAGCATCATCACGATTCC |
| Platte | CTGCAGCATCATCACGATTCC | CTGCAGCATCATCACGATTCC | CTGCAGCATCATCACGATTCC |
| Reeder | CTGCAGCATCATCACGATTCC | CTGCAGCATCATCACGATTCC | CTGCAGCATCATCACGATTCC |
| Rioblanco | CTGCAGCATCATCACGATTCC | CTGCAGCATCATCACGATTCC | CTGCAGCATCATCACGATTCC |
| SS_mpv57 | CTGCAGCATCATCACGATTCC | CCGCAGCATCATCACGATTCC | CTGCAGCATCATCACGATTCC |
| SY_Capstone | CTGCAGCATCATCACGATTCC | CCGCAGCATCATCACGATTCC | CTGCAGCATCATCACGATTCC |
| TA1615 | CTGCAGCATCATCACGATTCC | CCGCAGCATCATCACGATTCC | CTGCAGCATCATCACGATTCC |
| TA1718 | CTGCAGCATCATCACGATTCC | CCGCAGCATCATCACGATTCC | CTGCAGCATCATCACGATTCC |
| TAM111 | CTGCAGCATCATCACGATTCC | CCGCAGCATCATCACGATTCC | CTGCAGCATCATCACGATTCC |
| TAM112 | CTGCAGCATCATCACGATTCC | CCGCAGCATCATCACGATTCC | CTGCAGCATCATCACGATTCC |
| Ta1662 | CTGCAGCATCATCACGATTCC | CCGCAGCATCATCACGATTCC | CTGCAGCATCATCACGATTCC |
| UI_Platinum | CTGCAGCATCATCACGATTCC | CCGCAGCATCATCACGATTCC | CTGCAGCATCATCACGATTCC |
| Vida | CTGCAGCATCATCACGATTCC | CTGCAGCATCATCACGATTCC | CTGCAGCATCATCACGATTCC |
| W7984 | CTGCAGCATCATCACGATTCC | CCGCAGCATCATCACGATTCC | CTGCAGCATCATCACGATTCC |

**Table S5**. Primers used in this study.

| K3634Fw | CAGCATCTCTCTGCCGGCAC | AP2L-B2 | Kpn1 | wt 60+30pb | genotyping |
| --- | --- | --- | --- | --- | --- |
| K3634Rev | TCGGTATCGAACAGCCCCAAGTAGA**G**GTAC |  |  | mut 90pb |  |
| K2233Fw | CCGGGGGAGCTCCAAGTACA | AP2L-A2 | ssp1 | wt 114pb |  |
| K2233Rev | AGCTAGGAGGAGAGCGATGGTGGTAAATA |  |  | mut 80+30pb |  |
| K2236Fw | CGCAGCAGCAGCTGCAGCATCATCACGAAT | AP2L-A2 | EcoR1 | wt 115pb |  |
| K2236Rev | CTGGCAAGCTTAGGTGGGTCT |  |  | mut 85+30pb |  |
| TaAP2B KASP Wt | GAAGGTGACCAAGTTCATGCTAGCCGCAGCATCATCACG | AP2L-B2 |  |  |  |
| TaAP2B KASP Mut | GAAGGTCGGAGTCAACGGATTAGCCGCAGCATCATCACA |  |  |  |  |
| TaAP2B KASP common | TCTCGACGGATGGTCTCC |  |  |  |  |
| cDNA_K3634_Fw | CCGGGGCAGCTCCAAGTACC | to check mutant transcript | | | |
| cDNA_K3634_Rev | GCTCGAACTCCTCGGCGTAA |  |  |  |  |
| Nested-ap2-2mutant_Fw | GAGGCCGACATCAACTTCAG |  |  |  |  |
| Nested-ap2-2mutant_Rev | CGAGGCTGCCCCTCTTGTT |  |  |  |  |
| K2233Fw | CCGGGGGAGCTCCAAGTACA |  |  |  |  |
| cDNA_K2233_Rev | GCTCGAACTCCTCGGCGTAG |  |  |  |  |
| AP2L5Fw | GGCTTCTACCCGAACGTACA | qPCR | | | |
| AP2L5Rev | GGCGGTAGARAATCCTGATG |  |  |  |  |
| AP2L2Fw | TTCGATACCGAGGAGGAAGC |  |  |  |  |
| AP2L2Rev | GCTGGGATCGAAGTTTGTCA |  |  |  |  |
| AP2L7Fw | GGAGGACGAGCCTGACGTT |  |  |  |  |
| AP2L7Rev | GAAGGCCTCATCCAGATGCT |  |  |  |  |
| AP2L1Fw | ACAACCCATGCCACTCTTCT |  |  |  |  |
| AP2L1Rev | GTGTTGGTTGCTTGACGATG |  |  |  |  |
| Actin F | ACCTTCAGTTGCCCAGCAAT |  |  |  |  |
| Actin R | CAGAGTCGAGCACAATACCAGTTG |  |  |  |  |
| MADS4/TaPI-1Fw | AGATGCTGGAGGAGGAGCAC |  |  |  |  |
| MADS4/TaPI-1Rev | CGGCATCTGGGAAGTGAAAT |  |  |  |  |
| MADS16/TaAP3Fw | AAAATGTCGATGCCGCTCTC |  |  |  |  |
| MADS16/TaAP3Rev | CTCCTGGGAGTGCTTCACCT |  |  |  |  |
| MADS3/TaAG-2Fw | AACTTCCTGCAGGCGAACAT |  |  |  |  |
| MADS3/TaAG-2Rev | TACTGCTGGCCGAGCTGAAG |  |  |  |  |
| MADS58/TaAG-1Fw | AGACTGAAAGGGGGCAACAG |  |  |  |  |
| MADS58/TaAG-1Rev | GCAGGAAGGTTCTCGGATCA |  |  |  |  |
| MADS1/TaSEP-2Fw | GGAGCAAGAATTGCAGGATG |  |  |  |  |
| MADS1/TaSEP-2Rev | GCTASACTGCCCTCCGTCTT |  |  |  |  |
| MADS34/TaSEP-5Fw | GCAGCCAGAGCACTTCTTCC |  |  |  |  |
| MADS34/TaSEP-5Rev | GGCTGGTTCACATCCATGC |  |  |  |  |
| MADS5/TaSEP-6Fw | GGCGACAAAGAGCCAACAGT |  |  |  |  |
| MADS5/TaSEP-6Rev | TCCAACATCCTGGCAAGACA |  |  |  |  |
| MADS7/TaSEP-4Fw | CAGTTGGAGGAGAGCAACCA |  |  |  |  |
| MADS7/TaSEP-4Rev | AAGGGGGTGGAAGAATCCAT |  |  |  |  |
| MADS8/TaSEP-3Fw | CCAACTTGCTCGGCTACGAC |  |  |  |  |
| MADS8/TaSEP-3Rev | TGCGTTGTTTATCTGCTCCTG |  |  |  |  |
| VRN1-Fw | AAGAAGGAGAGGTCACTGCAGG |  |  |  |  |
| VRN1-Rev | GGCTGCACTGCCGCA |  |  |  |  |
| FUL2-FW | CCATACAAAAATGTCACAAGC |  |  |  |  |
| FUL2-Rev | TTCTGCCTCTCCACCAGTTC |  |  |  |  |
| FUL3-Fw | ATGGATGTGATTCTTGAACG |  |  |  |  |
| FUL3-Rev | AGTTGCCTTTGACTCTTCTG |  |  |  |  |
