# Supplementary material for: APETALA 2‐like genes AP2L2 and Q specify lemma identity and axillary floral meristem development in wheat
Source: Plant J. 2019 Oct 15;101(1):171–87. doi: 10.1111/tpj.14528 (PMC6972666; doi:10.1111/tpj.14528)
Supplement: Supplementary file 3 [file TPJ-101-171-s003.docx]

**TPJ-00703-2019.R1_Supporting_Information_Legends**

**Tables**

**Table S1**. **Locus name for the different wheat *AP2*-like genes**. Wheat homeologs harboring a miR172 target site in the Wheat Genome RefSeqv1.1 and their closest barley, *Brachypodium distachyon* and rice orthologs.

**Table S2. Reciprocal BLASTN searches for wheat homologs of *SNB/SID1****.* The *Brachypodium* gene *Bradi1g53650* was used as query*.* Note that in rice and *B. stacei* the reciprocal BLASTN returns the correct *Bradi1g53650.* Whereas the closest homologs in *T. urartu* (progenitor of genome A in polyploid wheat), einkorn wheat (genome A^m^), *A. tauschii* (progenitor of genome D in hexaploid wheat), wild emmer Zavitan, rye, and barley, return the *Brachypodium* orthologs of *AP2L5* (blue), *AP2L1* (green), *AP2L7* (orange), and *AP2L2* (red). AP2-like genes of the SNB/SID1 clade are highlighted in gray.

**Table S3**. Wheat orthologs of MIKC-type MADS-box genes involved in the ABCE flowering model.

**Table S4**. Natural variation in miR172 target site of *AP2L2*.

**Table S5**. Primers used in this study.

**Figures**

**Figure S1**. ***AP2L2* induced mutants.** **(A)** Genomic structure of *AP2L2* and *AP2L5* genes showing the position of the Kronos mutations K2233, K3634 and K3946. Exons are in blue, AP2 domains in pink, and the miR172 target site in red. Mutations in the A homeolog are shown above the gene structure and in the B homeolog below. The *AP2L-5B* homeolog has a natural 2 nt deletion. **(B)** Effect of the K2233 mutation in the splicing of *AP2L-2A* homeolog. The correct (wt) and mutant (K2233) splicing of the 4^th^ and 5^th^ exons are shown below the genomic structure. The K2233 line has a G-to-A change (red) in the splicing donor site of the 4^th^ intron. That mutation results in the use of a nearby GT site and retention of 4 extra intronic nucleotides (underlined). **(C)** Grain number per spike in K3634 homozygous mutant segregating for the K2233 mutation. MW= K3634 mutant/ K2233 wild type; MH= K3634 mutant/ K2233 heterozygous; MM= K3634 mutant/ K2233 mutant. Note the reduced number of grains in the double *ap2l-2A* *ap2l-2B* mutant (MM). **(D-E)** Dissected lodicules from wild type **(D)** and *ap2l2* **(E)** florets, scale bar = 0.5mm. **(F)** Swollen area in lodicules from wild type (Wt) and *ap2l2* florets. Bars represent mean ± s.e.m. (based on 20 lodicules from 10 florets). *** indicates statistically significant differences (*P* < 0.001) by *t*-test.

**Figure S2.** **Spikelet number and heading time for wild type and *ap2l2*, *ap2l5* and *ap2l2* *ap2l5* mutants.** **(A)** Spikelet number in the primary spike and **(B)** days to heading for wild type Kronos (Wt), *ap2l2*, *ap2l5* and *ap2l2* *ap2l5* mutants. Bars represent mean ± s.e.m. and different letters above the bars indicate statistically significant differences (P<0.05) by Student–Newman–Keuls test.

**Figure S3**. **Scanning electron microscopy images of dissected apices from wild type (Wt) and *ap2l2*, *ap2l5* and *ap2l2* *ap2l5* mutants.** **(A)** Spike development at Waddington stage W3-3.25. Scale bars are 100 µm **(B)** Spike development at Waddington stage W4.5-5 stages. Scale bars are 500 µm.

**Figure S4**. **Transcript levels of wheat MADS-box genes involved in floral organ identity during spike development.** Based on previously published RNASeq dataset (Li et al., 2018, Scientific Reports 8:15338). **(A)** Class A genes: *VRN1*, *FUL2* and *FUL3*. **(B)** Class B genes: *TaPI1*and *TaAP3*. **(C)** Class C genes: *TaAG2* and *TaAG1*. **(D)** Class E genes expressed earlier in spikelet development: *TaSEP5* (*= PAP2*) and *TaSEP6*. **(E)** Class E genes expressed later in spikelet development: *TaSEP2*, *TaSEP8* and *TaSEP7*. FPKM= Fragments per kb of transcript per million mapped reads. W1.0 = vegetative stage, W1.5 = elongating apex, W2.0 = early double ridge stage, W2.5 = double ridge stage, W3.0 = glume primordium differentiation stage, W4.0 = floret primordium differentiation stage.

**Figure S5.** **Mutation in the miR172 binding site of *AP2L-B2* in hexaploid wheat (*rAp2l-B2*).** **(A)** Picture showing a representative penultimate spikelet from a wild type (WT) spike and a homozygous mutant (Mut). Red arrows point to glume 1 (G1) and glume 2 (G2) awn tips, scale bar= 1cm. **(B-C)** Length of the awn of the first **(B)** and second **(C)** glumes in the penultimate spikelet of F_2_ plants segregating for *rAp2l-2B* mutation (Wt = homozygous wild type, Het = heterozygous, and Mut = homozygous mutant plants) (n ≥ 10). Different letters above the bars indicate statistically significant differences (*P* < 0.05) by Student–Newman–Keuls test. **(D)** Picture of lodicule (left) and graph of average lodicule swollen area (right) from F_2_ plants segregating for the *rAp2l-2B* mutation (Wt = homozygous wild type, and Mut = homozygous *rAp2l-2B*) (n≥16). *** = *P* < 0.0001. In all graphs bars represent ± s.e.m.
